# Hypoxic Characteristic Genes Predict Response to Immunotherapy for Urothelial Carcinoma

**DOI:** 10.3389/fcell.2021.762478

**Published:** 2021-11-25

**Authors:** Shuo Hong, Yueming Zhang, Manming Cao, Anqi Lin, Qi Yang, Jian Zhang, Peng Luo, Linlang Guo

**Affiliations:** ^1^ Department of Oncology, Zhujiang Hospital, Southern Medical University, Guangzhou, China; ^2^ Department of Pathology, Zhujiang Hospital, Southern Medical University, Guangzhou, China

**Keywords:** metastatic urothelial carcinoma, prognosis model, immune checkpoint inhibitors, hypoxia, immune microenvironment

## Abstract

**Objective:** Resistance to immune checkpoint inhibitors (ICIs) has been a massive obstacle to ICI treatment in metastatic urothelial carcinoma (MUC). Recently, increasing evidence indicates the clinical importance of the association between hypoxia and immune status in tumor patients. Therefore, it is necessary to investigate the relationship between hypoxia and prognosis in metastatic urothelial carcinoma.

**Methods:** Transcriptomic and clinical data from 348 MUC patients who underwent ICI treatment from a large phase 2 trial (IMvigor210) were investigated in this study. The cohort was randomly divided into two datasets, a training set (*n* = 213) and a testing set (*n* = 135). Data of hypoxia-related genes were downloaded from the molecular signatures database (MSigDB), and screened by univariate and multivariate Cox regression analysis to construct a prognosis-predictive model. The robustness of the model was evaluated in two melanoma cohorts. Furthermore, an external validation cohort, the bladder cancer cohort, from the Cancer Genome Atlas (TCGA) database, was t used to explore the mechanism of gene mutation, immune cell infiltration, signaling pathway enrichment, and drug sensitivity.

**Results:** We categorized patients as the high- or low- risk group using a four-gene hypoxia risk model which we constructed. It was found that patients with high-risk scores had significantly worse overall survival (OS) compared with those with low-risk scores. The prognostic model covers 0.71 of the area under the ROC curve in the training set and 0.59 in the testing set, which is better than the survival prediction of MUC patients using the clinical characteristics. Mutation analysis results showed that deletion mutations in RB1, TP53, TSC1 and KDM6A were correlated with hypoxic status. Immune cell infiltration analysis illustrated that the infiltration T cells, B cells, Treg cells, and macrophages was correlated with hypoxia. Functional enrichment analysis revealed that a hypoxic microenvironment activated inflammatory pathways, glucose metabolism pathways, and immune-related pathways.

**Conclusion:** In this investigation, a four-gene hypoxia risk model was developed to evaluate the degree of hypoxia and prognosis of ICI treatment, which showed a promising clinical prediction value in MUC. Furthermore, the hypoxia risk model revealed a close relationship between hypoxia and the tumor immune microenvironment.

## Introduction

Metastatic urothelial carcinoma (MUC) is a common malignancy which occurs in the urothelial organs of the urinary system (Humphrey et al., 2016; Moch et al., 2016). According to a Global Data survey, bladder cancer ranks as the 10th most common cancer in the world, and it is four times more common in males than females (Bray et al., 2018). At present, platinum-based chemotherapy is the first-line treatment for patients with metastatic urothelial carcinoma (Flaig et al., 2020; Witjes et al., 2021). However, it is not uncommon for chemotherapy to fail or for a positive treatment response to only be short term (Koshkin and Grivas, 2018). Therefore, in recent years, new focus has been given to developing treatments that use immune checkpoint inhibitors (ICIs).

At present, some known immune checkpoint inhibitors, including antibodies against cytotoxic T lymphocyte associated protein 4 (CTLA-4) as well as programmed cell death 1 (PD-1) receptor and its ligand (PD-L1), have displayed promising efficacy in metastatic urothelial carcinoma (MUC) (Darvin et al., 2018; Bagchi et al., 2021). According to the 2020 guidelines of the European Association of Urology (EAU), pembrolizumab and atezolizumab have become the first-line treatment option for locally advanced or metastatic urothelial carcinoma after failure of platinum chemotherapy (Witjes et al., 2021). In a phase III clinical trial involving 542 patients with advanced MUC after platinum chemotherapy, KEYNOTE-045 showed that the median overall survival (mOS) of the pembrolizumab group was longer than that of the second-line chemotherapy group by 3 months (10.3 vs 7.4 months; *p* = 0.002) (Bellmunt et al., 2017). Long term results from the same phase III trial (>2-year follow-up) indicated that pembrolizumab had longer 1-year and 2-year overall survival (OS) results than chemotherapy (Fradet et al., 2019). However, only part of the population can benefit from immunotherapy. In a phase II, multicenter, uncontrolled trial of nivolumab for platinum-resistant advanced urothelial carcinoma, the objective response rate was 19.6% in 265 patients (Sharma et al., 2017; Powles et al., 2018). A single-arm phase II trial evaluated the efficacy of pembrolizumab as the first-line treatment in 370 patients with advanced urothelial carcinoma who were not suitable for cisplatin based therapy. Only 5% of patients achieved complete remission (Balar et al., 2017). Thus, exploring the mechanism of immunosuppressant in patients with metastatic urothelial carcinoma and seeking accurate predictive biomarkers is warranted.

Due to the rapid growth and abnormal proliferation of solid tumors, inadequate oxygen supply causes the tumor microenvironment to experience uneven blood distribution and immunosuppression (Choudhry and Harris, 2018; Krzywinska and Stockmann, 2018). Furthermore, the hypoxic microenvironment of the tumor prevents adequate oxidation of glucose, promotes tumor cells to derive energy not only from oxidative phosphorylation but also from glycolysis, and results in the accumulation of lactic acid and adenosine, that lead to abnormal T cell ratio and T cell dysfunction (Beavis et al., 2015). A growing number of studies have revealed that the hypoxic microenvironment reduces the stimulation of T cells, increase the recruitment of immunosuppressive cells such as regulatory T (Treg) cells, and reduce the mobility of antigen-presenting cells, monocytes, and dendritic cells (Ohta, 2016; Yuen and Wong, 2020). Another study suggests that poor prognosis caused by the immune-desert-type colon cancer may be associated with tumor hypoxia (Craig et al., 2020). However, there is a lack of research focused on hypoxic microenvironments in immunotherapy for patients with MUC.

In this study, we constructed a prognostic risk model to evaluate the impact of hypoxia on the efficacy of immune checkpoint inhibitor (ICI) therapy in patients with MUC. In addition, we explore the possible mechanisms of hypoxic microenvironments and the efficacy of ICI in patients with MUC by looking at biological markers, tumor immune typing, gene mutation, and immune microenvironment.

## Methods

### Data Collection and Preprocessing

Genomic, transcriptomic, and clinical data of patients with metastatic urothelial carcinoma treated with an anti-PD-L1 drug (Atezolizumab) were downloaded from a study conducted by Mariathasan et al. (2018). We split the data into a training set and a testing set, with 60% of samples for the training set, and the remaining 40% of samples for the testing set according to hierarchical random grouping. These groupings were used to construct and evaluate the prognostic model for risk of oxygen deficiency. From the Memorial Sloan Kettering Cancer Center (MSKCC) database, a cohort of cutaneous skin melanoma (MSKCC skcm) patients receiving anti-CTLA-4 immunotherapy was recorded in the data published by Samstein et al. (Snyder et al., 2014). We also downloaded the transcriptomic data for PD-1 treatment in the GSE78220 data set from the Gene Expression Omnibus (GEO) database (Hugo et al., 2016) to further analyze the prognostic effect of the hypoxia risk model.

The “TCGAbiolinks” R package (Colaprico et al., 2016) was used to download the TCGA BLCA bladder cancer data set (*n* = 412) from The Cancer Genome Atlas (TCGA) database. This data set included clinical information, genomic data, and transcriptomic data. Patients with metastatic urothelial carcinoma were extracted for subsequent analysis (Cancer Genome Atlas Research, 2014; Robertson et al., 2017). The GSE120736 bladder cancer data set was downloaded from the GEO database to analyze the immune microenvironment.

We downloaded the GSE158632 dataset from the GEO database for subsequent functional validation analysis (Nersisyan et al., 2021). The GSE158632 dataset provides high-throughput sequencing data of 18 samples of Caco-2 and HT-29 cells, nine from the CACO-2 cell lines and nine from the HT-29 cell lines. Each cell line contains nine samples, among them, three samples are control groups and the other six samples are hypoxia groups.

### Identification of Hypoxia-Related Genes Impacting Prognosis and Construction of the Risk Prognostic Model

We collected the hallmark gene sets (Liberzon et al., 2011) from the molecular signatures database (MSigDB) (Liberzon et al., 2015). The single sample pathway enrichment (ssGSEA) score of the Hallmark gene sets of all samples in the MUC cohort was calculated using the “GSVA” R package (Hänzelmann et al., 2013). The relationship of the ssGSEA score of Hallmark gene sets and ICI efficacy and survival was analyzed using the “LIMMA” and “survival” and “survimner” R packages. Then, the “survival” (https://github.com/therneau/survival) and “survminer” (https://rpkgs.datanovia.com/survminer/) R packages were used to identify genes related to overall survival in the hypoxia gene sets in the MUC cohort. Based on the 44 genes screened as previously described, we established a four-gene hypoxia risk score model for risk stratification using univariate and multivariate Cox regression for the MUC training set.

$$Risk\;score=\sum_{n=1}^{n}\left(coefficient*gene\;expression\right)$$

where N = 4, gene expression was the expression value of hypoxia genes, and the coefficient was the corresponding multivariable Cox regression coefficient.

A Kaplan–Meier survival analysis and the log-rank test were performed to evaluate the difference of patient survival time. According to median overall survival, patients with metastatic urothelial carcinoma were divided into two groups. Patients with melanoma were grouped into two groups according to the best cutoff point calculated by the function “surv_cutpoint” of the “survminer” R package. The receiver operating characteristic (ROC) curve was used to analyze the sensitivity and specificity in the MUC training and testing sets.

### Analysis of Driver Gene Mutation

A list of driver genes was taken from the cancer gene census (CGC) database, and panoramic maps of driver gene mutations were generated by “ComplexHeatmap” R package (Gu et al., 2016) for the MUC training set, MUC testing set, and TCGA BLCA set. The top 20 driver genes were selected for the MUC training set and the MUC testing set. As for the TCGA BLCA queue, we selected the top 20 driver genes and the other top 20 driver genes in the two queues of MUC to analyze. The “Maftools” R package (Mayakonda et al., 2018) was used for mutual exclusion analysis of the driver mutation genes in the above cohorts.

### Analysis of the Immune Microenvironment

Gene length was calculated using the “TxDb.Hsapiens.UCSC.hg38.knownGene” (https://bioconductor.org/packages/) R package. Raw count gene expression data of the MUC and TCGA BLCA data set and the GSE120736 data set were standardized and converted into the transcripts per million (TPM) data format. The “xCell” R package was utilized to evaluate the infiltration abundance of immune cells in the tumor microenvironment (TME) (Aran et al., 2017). Differences were considered significant with a *p*-value less than 0.05. Differential expression analysis in immune-related genes between the MUC and TCGA BLCA data sets was performed using the “LIMMA” R package (Ritchie et al., 2015). The list of immune gene sets was sourced from research by Thorsson et al. (2018) and Hao et al. (2018).

### Pathway Enrichment Analysis

The “clusterProfiler” R package (Yu et al., 2012) was used to conduct gene ontology (GO) analysis (|logFC > 0|, *p* 0.05) as well as gene set enrichment analysis (GSEA) (Subramanian et al., 2005) (|logFC > 0|). Gene sets for GSEA enrichment analysis were downloaded from the Hallmark gene sets (H), curated gene sets (C2), and ontology gene sets (C5) in the molecular signatures database (MSigDB).

### Statistical Analysis

Overall survival was estimated using the Kaplan–Meier method, and the difference between the high- and low- risk groups was examined using log-rank test in each data set. The association of risk scores with tumor mutational burden (TMB) and tumor neoantigen burden (TNB) was analyzed using Pearson correlation analysis. Wilcoxon’s test was used to test the risk score difference between TCGA phenotype and immune phenotype. The risk scores were then compared between the immune phenotypes through Kruskal-Wallis test. Associations between risk scores and ICI efficacy or driver gene mutation frequency were assessed, using Fisher’s exact test. The “LIMMA” R package was used to analyze the differential genes in the high- and low- risk score groups (*p* < 0.05). All analyses were performed using R software (v4.0.5), and the *p*-value was bilateral.

## Results

### Establishment of the Hypoxia Signature Model

**FIGURE 1:**
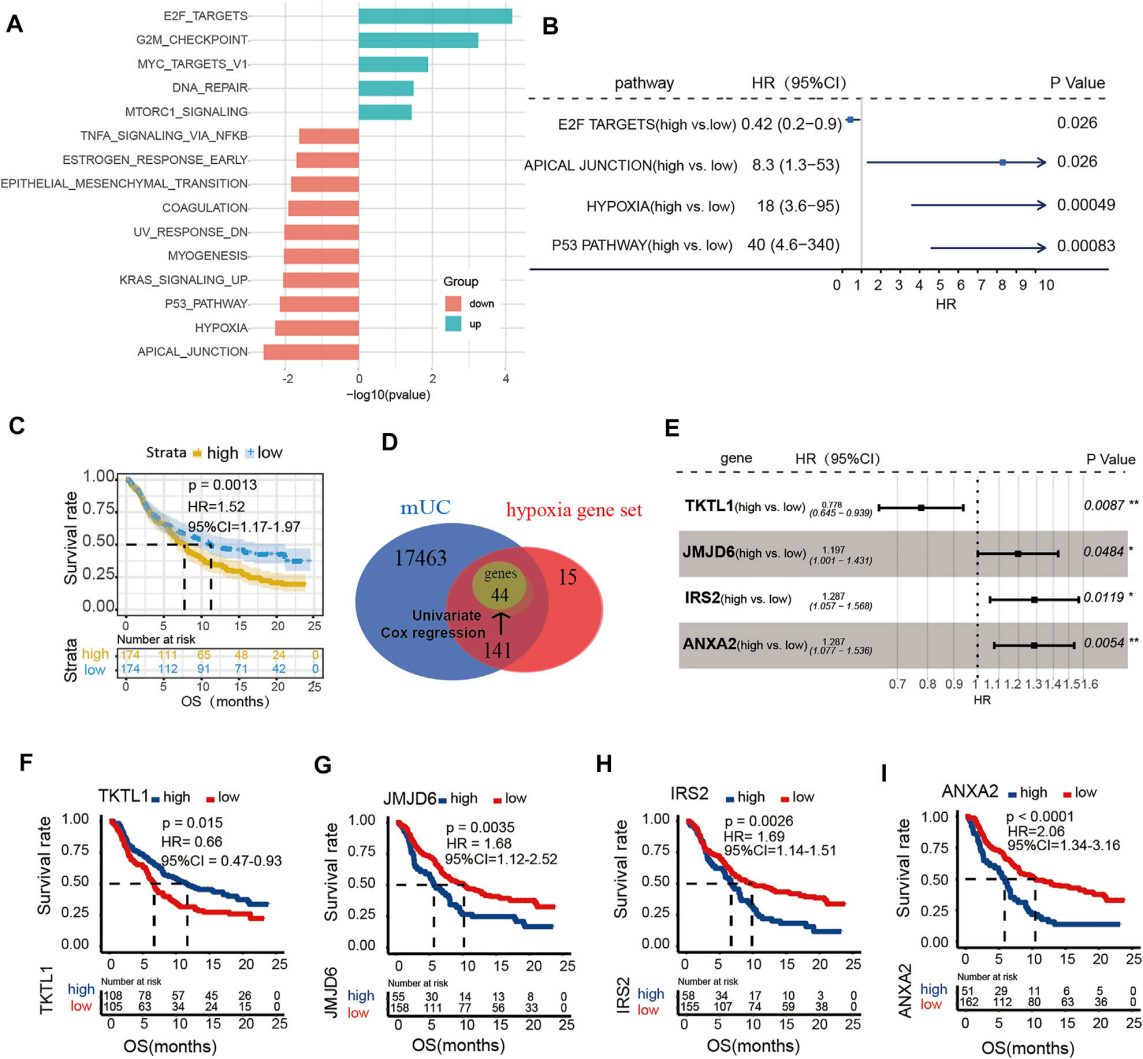
The expression of hypoxia microenvironment in metastatic urothelial carcinoma (mUC). **(A)** Histogram showed that the ssGSEA score of the Hallmark pathway set was different in CR/PR vs PD/SD patients (logFC < 0, *p* < 0.05). **(B)** The enrichment scores of hypoxia gene set were different in forest map. **(C)** KM curve showed that patients with high score of hypoxia gene set had poor OS (log rank *p* < 0.05). **(D)** The gene in each data set was shown by Wayne map. The forest map shows the genes and coefficients of risk score.

To investigate the role of hypoxia in ICI treatment, we calculated ssGSEA scores of the 50 pathways set from the GSEA Hallmark gene set. We found that genes in the hypoxia pathway was upregulated in patients without response (*p* < 0.05, Figure 1A). Meanwhile, results of univariate Cox regression revealed that high score of hypoxia ssGSEA was correlated with poor prognosis in patients with MUC (*p* = 0.00049, HR = 18, 95% CI = 3.6–95, Figure 1B). Kaplan-Meier survival curves, illustrated that patients with poor prognosis had high expression of hypoxia signature genes (log-rank *p* = 0.0013, HR = 1.52, 95% CI = 1.17–1.97, Figure 1C). Univariate analysis identified forty-four genes significantly related to overall survival in the Hallmark hypoxia gene set as potential prognostic factors for further analysis (Supplementary Figure S1B). Next, four hypoxia genes, including TKTL1, JMJD6, IRS2 and ANXA2, were identified as independent prognostic factors for OS by multivariate analysis (Figure 1E). These identified prognostic factors were applied as basic indexes in the novel prognostic model. Finally, we developed a hypoxia risk score model to predict OS based on a low-risk hypoxia gene (TKTL1) and three high-risk genes (JMJD6, IRS2, ANXA2) (Figures 1F–I).

### Validation of the Hypoxia Risk Prognostic Model

**FIGURE 2:**
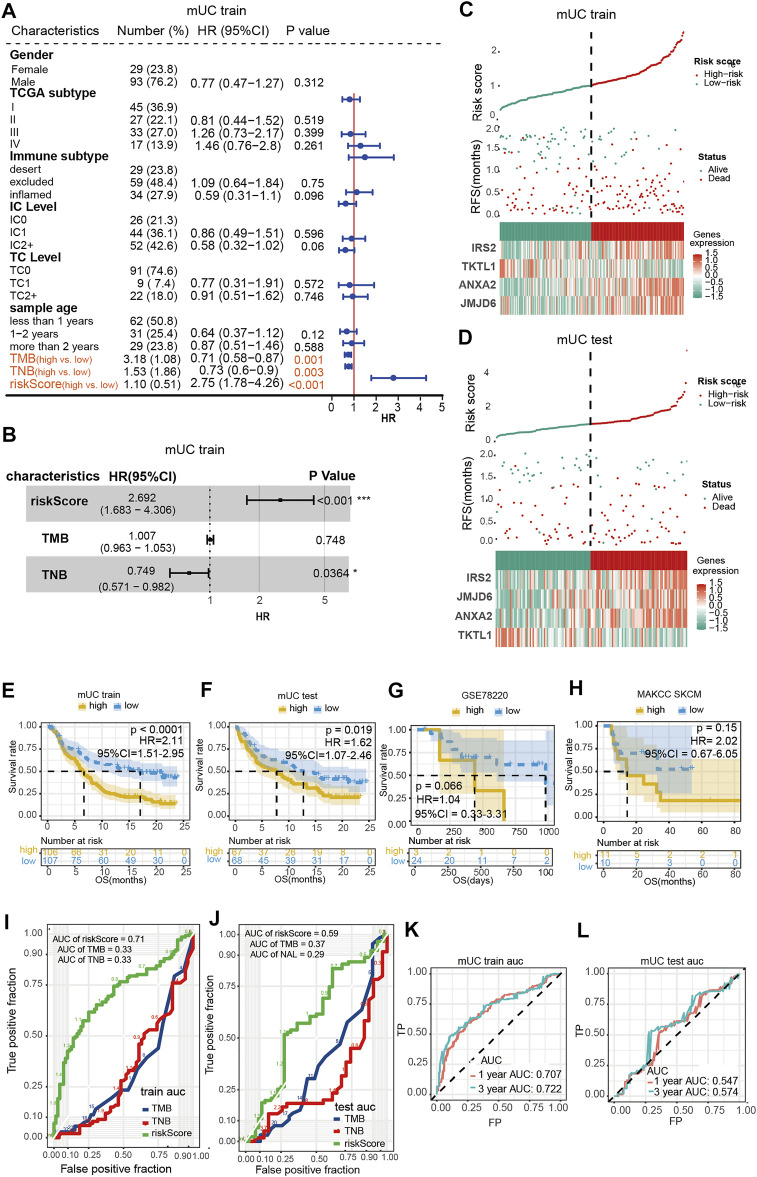
Evaluation of hypoxia risk prognosis model. **(A,B)** shows the results of a univariate Cox regression and multivariate Cox regression showed that risk score was an independent prognostic factor for patients with mUC treated by ICIs. **(C,D)** Risk curve showed the relationship between gene expression, hypoxia risk score and survival. **(E,H)** Kaplan Meier curve showed that the high scores of hypoxia risk were associated with poor prognosis in MUC training set, testing set, MSKCC skcm and GSE78220 data sets. **(I,L)** ROC curve evaluated the AUC values of risk score, TMB and TNB in the training set and testing set queue, and the training set had better prediction effect (AUC = 0.71). ROC curve was used to evaluate the AUC value of risk score predicting 1-year and 3-year survival rate in the training set and testing set cohort.

To further evaluate the performance of the hypoxia model, we compared our hypoxia prognostic model with the routine clinical prognostic characteristics to ascertain whether our risk assessment model was a feasible prognostic tool for MUC patients. We subjected the predictor variables of hypoxia risk score, clinical signatures, TMB, and TNB of patients in the MUC training set to univariate Cox regression analyses. It was found that three factors, including hypoxia risk score, TMB, and TNB, were significant (*p*-value < 0.05) (Figure 2A). Next, we found that the hypoxia risk scores (HR = 2.75, 95% CI = 1.683–4.306, *p* < 0.001) and TNB (HR = 0.73,95% CI = 0.571–0.982, *p* = 0.0364) (Figure 2B) were independent predictors for ICIs treatment efficacy in patients with MUC. Collectively, the above results indicated that the hypoxia risk score model was a favorable predictor of ICIs treatment efficacy (HR = 2.692, 95% CI = 1.683–4.306, *p* < 0.001).

The training and testing set risk curves (Figures 2C,D) also confirmed the accuracy of the model. Patients were categorized into the high- and low-risk groups according to the median hypoxia risk score, and analyzed with Kaplan–Meier survival curves. As shown in Figures 2E,F, patients with high-risk scores had shorter overall survival time than those with low-risk scores (log-rank *p* < 0.0001, HR = 2.11, 95% CI = 1.51–2.95; log-rank *p* < 0.0001, HR = 2.11, 95% CI = 1.51–2.95; *p* <0.0001; log-rank *p* = 0.019, HR = 1.62, 95% CI = 1.07–2.45). Although no significant difference was observed across groups in the MSKCC skcm cohort treated with anti-CTLA-4 drug or the GSE78220 melanoma treated with anti-PD-1 drug, patients with high prognostic scores showed a tendency of short-term OS (Figures 2G,H).

The area under the ROC curve (AUC) of three independent prognostic factors: hypoxia risk score, TMB, and TNB, was calculated to evaluate the efficacy of the prediction model. The area under the curve (AUC) for the hypoxia-based model (AUC = 0.71) was higher than that of both the TMB and TNB based prediction (AUC = 0.33, 0.33) in the MUC training set. Similar tendencies were observed in the testing group, in which the areas under the curve (AUC) of hypoxia risk score, TMB, and TNB were 0.59, 0.37 and 0.29, respectively (Figures 2I,J). Furthermore, we found that the hypoxia risk score model had the best predictive value at 3 years (training set: 1-year AUC = 0.707, 3-year AUC = 0.722, Figure 2K; test set: 1-year AUC = 0.547, 3-year AUC = 0.574, Figure 2L).

### Prognostic Model and Clinical Features of Hypoxia Risk

Next, we evaluated the possible associations between hypoxia scores and clinical characteristics. We investigated the relationship among the following indicators: hypoxia scores, immunotherapy efficacy, PD-L1 expression level on the tumor surface, PD-L1 expression level in immune cells, immunogenicity related factors, TCGA molecular phenotype, and tumor immune phenotype.

**FIGURE 3:**
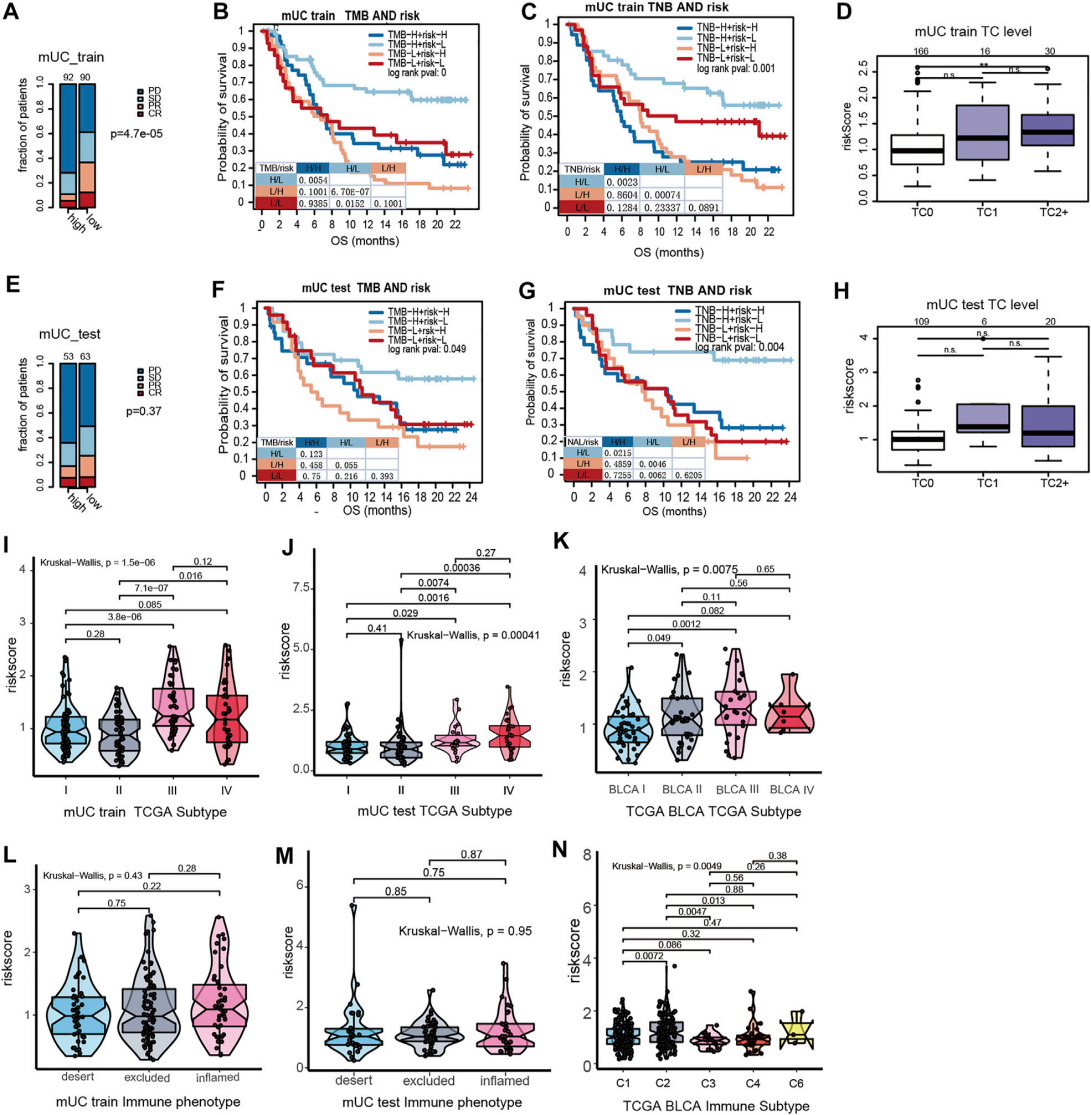
The interations of clinical features in MUC and hypoxia risk score. **(A)** The stacked histogram showed that the number of non-responders to ICI in the MUC Training set high risk score subgroup was higher than that in the low score subgroup. **(B,C)** Kaplan Meier curve showed the survival results of the joint analysis of MUC Training set risk score with TMB and TNB. **(D)** Box plot showed that the PD-L1 expression in tumor cells of the MUC training set. PD-L1 was higher in tumor cells with high hypoxia risk score. **(E)** Histogram showed that the number of non-responders to ICI in the MUC testing set high risk score subgroup was higher than that in the low score subgroup. **(F,G)** KM curve showed the survival results of risk score combined with TMB and TNB in the MUC testing set. **(H)** Box plot showed the expression of risk score and PD-L1 in tumor cells of the MUC testing set. There was no significant difference in the expression level of risk score among different PD-L1 expression levels. **(I–K)** Violin diagram shows the distribution of MUC training set, MUC testing set and TCGA BLCA risk score in TCGA molecular subtypes. The results showed that the risk score of TCGA III was higher than that of other types. The distribution of the MUC training set, MUC testing set and TCGA BLCA risk score in immunophenotyping was shown by violin diagram.

As shown in Figure 3, patients with high-risk scores had an inadequate response to anti-PD-L1 inhibitors (*p* = 0.000047, Figure 3A). Despite no statistical difference in the testing set, patients with high-risk scores illustrated a trend of poor response to ICI treatment (*p* = 0.37, Figure 3B). We further performed survival analysis on hypoxia risk scores, TMB, and TNB, to explore the relationship between the survival rate and prediction factors that may affect the efficacy of ICIs. We found that high-TMB and low-risk score patients have longer OS (paired log rank test *p* = 0.00000067). Significantly increased OS was observed in high-TNB and low-risk patients compared with low-TNB and high-risk patients (paired log-rank test *p* = 0.00074, Figures 3B,C). In the MUC testing set, the high-TMB and low-risk group showed a similar trend of association with OS in the training set (paired log-rank test *p* = 0.055). Additionally, patients with high TNB and a low hypoxia risk score had longer OS (paired log-rank test, *p* = 0.046, Figures 3F,G). These results suggest that the risk score of hypoxia combined with characteristics of tumor immunogenicity are good predictors of survival and the efficacy of anti-PD-L1 inhibitors.

According to previous studies, the MUC cohort was divided into three grades according to the results of the PD-L1 immunohistochemical staining of tumor cells. TC0 indicated that the level of PD-L1 was lower than 1%, TC1 was between 1 and 5%, and TC2+ was more than 5%. The results showed that the hypoxia risk score of TC2+ was significantly higher than that of TC0 (Figure 3D). However, in the MUC testing set, there was no significant difference in the hypoxia risk score at different TC levels (Figure 3H). Based on the results of immune cell PD-L1 staining, the MUC cohort was also divided into three grades. IC0 indicated that the PD-L1 level was lower than 1%, IC1 indicated that the PD-L1 expression level was between 1 and 5%, and IC2+ indicated that the PD-L1 expression level was more than 5%. There was no significant difference in PD-L1 expression between the MUC training set and the testing set on hypoxia risk scores (Supplementary Figures S3C,F).

In a study by Thorson et al. (Ritchie et al., 2015), the TCGA BLCA data set was classified into six subtypes according to tumor molecular characteristics. These were C1 (wounding healing) and C2 (IFN-γ Dominant), C3 (Inflammatory), C4 (Lymphocyte Depleted), C5 (Immunologically Quiet), and C6 (TGF-βDominant). The results show that the risk scores of hypoxia for the C2 type are the highest among all immune subtypes (Figure 3J). The TCGA phenotype is based on the TCGA molecular phenotype of bladder cancer (Cancer Genome Atlas Research, 2014). We observed that the TCGA type III (basic/square-like) had a high hypoxia risk score, and overexpression of KRT14 and KRT5 proteins (Figures 3I,K).

### Hypoxia Risk Prognostic Model, Gene Mutation, Co-Occurrence and Mutual Exclusion Analysis of Mutated Genes

**FIGURE 4:**
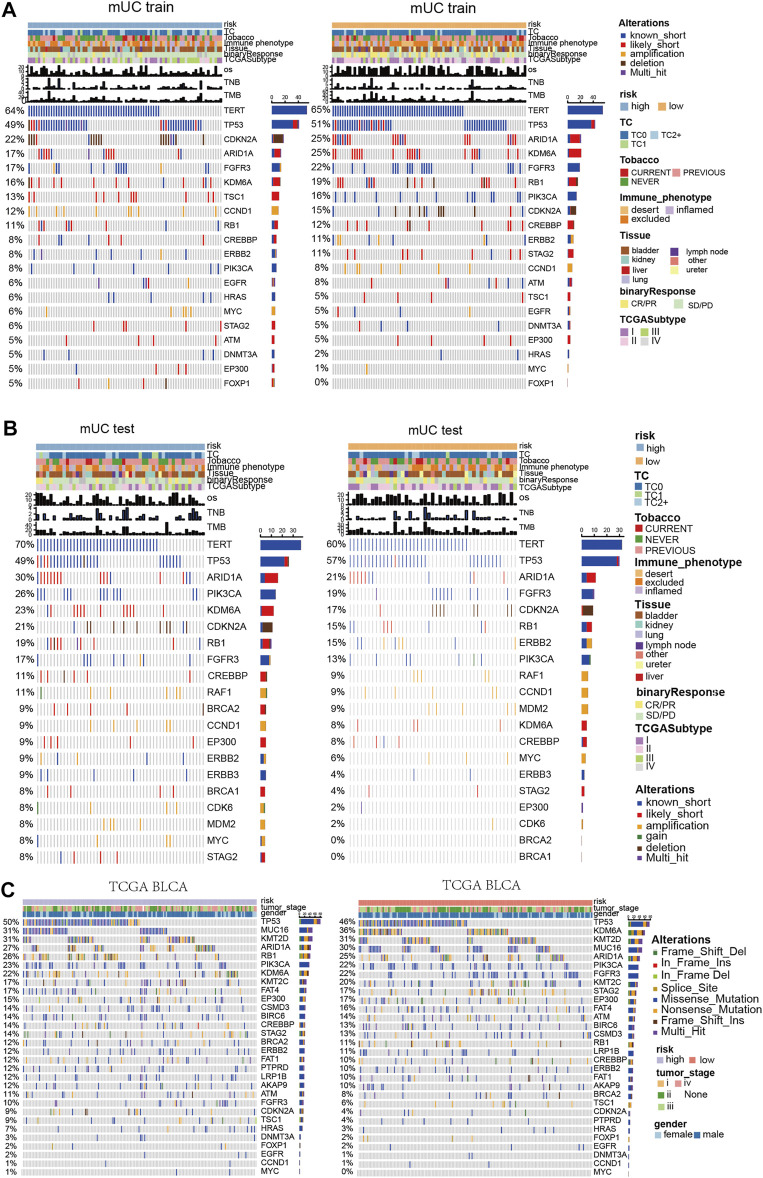
Panoramic view of metastatic urothelial carcinoma and TCGA BLCA mutation gene, gene co mutation and mutual exclusion analysis. **(A)** Panoramic view of the top20 driving gene mutation of the MUC training set. **(B)** Panorama of top20 driving gene mutation of the MUC testing set. **(C)** Panorama of top driving gene mutation in the TCGA BLCA cohort.

Previous studies have shown that the chronic hypoxic microenvironment reduces DNA repair ability and impairs the DNA repair system. Therefore, we further distinguish the function of hypoxic microenvironment on gene mutations in metastatic urothelial carcinoma (Figures 4A–C). In the MUC training set, TERT (64 vs 65%) and TP53 (49 vs 51%) were in the high- and low- risk groups, among which TERT and TP53 were known to have short mutations as the major mutation. The mutation frequencies of ARID1A, KDM6A, FGFR3, and Rb1 were low in the subgroup with high-risk scores. Notably, there was no significant difference in the mutation frequency of these genes in any group.

In the MUC testing set, TERT (70 vs 60%) and TP53 (49 vs 57%) had high frequencies of known short mutations in both the high-risk and low-risk subgroups. However, in contrast to the MUC training group, the mutation frequency of ARID1A, KDM6A, and Rb1 was higher in the subgroup with high-risk scores. There was no significant difference in the mutation frequency of these genes in any group. In the TCGA BLCA cohort, Rb1, KDM6A, and FGFR3 showed significant differences in mutation frequency among the hypoxia risk groups. The high-risk group had a higher mutation frequency of Rb1 (26%) than that of the low-risk subgroup (11%), whereas the high-risk group had lower mutation frequencies of KDM6A (22%) and FGFR3 (10%) than that of the low-risk subgroup.

Diagrams of mutation genes with computation and mutual exclusion analysis (Supplementary Figures S4A–C) show the results of three clinical cohorts. In the high-risk MUC training set, RB1 and TP53, ERB2 and TSC1, Rb1 and ARID1A had co-mutations. In the low-risk set, TP53 was mutable with FGFR3 and CDKN2A, TSC1 and EP300, Rb1 and ERBB2, PIK3CA and STAG2. In the TCGA BLCA data set with high hypoxia risk scores, Rb1 and TP53, KDM6A, and FGFR3 had co-mutations. TP53 and FGFR3 had mutually exclusive mutations. In the TCGA BLCA cohort with low hypoxia risk scores, Rb1 showed co-mutation with TP53 and ARID1A, and Rb1 had a mutually exclusive mutation with FGFR3.

### Immune Correlation Analysis

Some studies suggest that a hypoxic microenvironment might affect the activation of infiltrating immune cells and the immune response of tumor cells. Therefore, we examined the possible associations between hypoxia and immune microenvironment. We used the xCell method to calculate the infiltration of immune cell components in the training set, testing set, TCGA BLCA cohort, and GSE120736 cohort downloaded from the GEO database.

**FIGURE 5:**
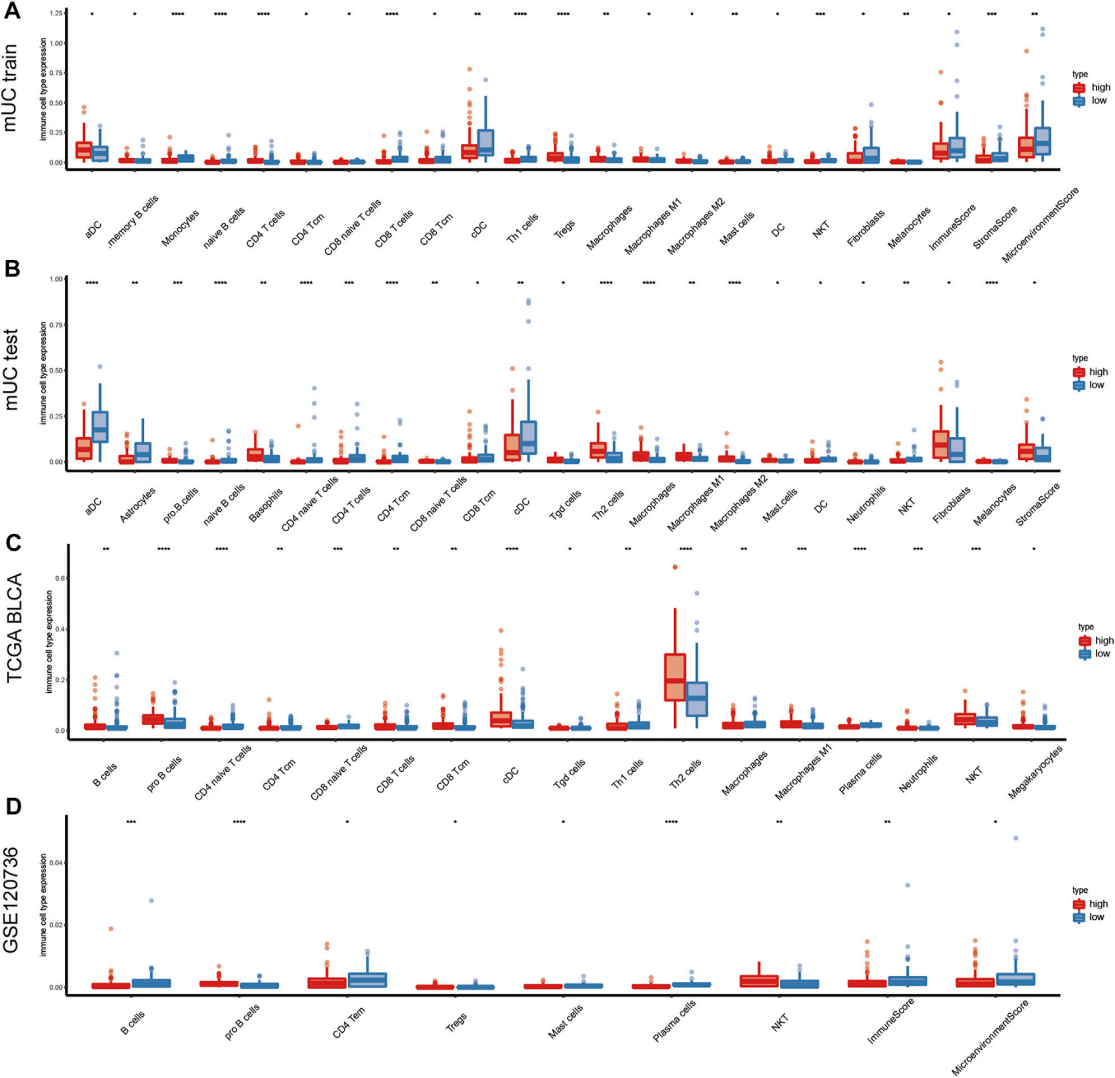
Changes of immune components in tumor hypoxia microenvironment. **(A–D)** Diagram shows the immune microenvironment infiltration score of the training set, testing set, TCGA BLCA and GSE120736 datasets in metastatic urothelial carcinoma. The figure shows the immune cells with statistical difference in the analysis results (*p* < 0.05), and the results show that T cells, B cells, macrophages, and Treg cells were more infiltrated in the high hypoxia risk score subgroup. (CD4 + Tcm: CD4^+^ central memory T cells, CD8 + Tcm: CD8^+^ central memory T cells, NKT: natural killer T cells).

As is shown in Figure 5, we found that the number of CD4^+^ T cells was higher in the high-risk subgroup than in the low-risk subgroup. And the immunosuppression-related cells, such as Treg cells and macrophages, were significantly higher in the high-risk subgroup than in the low-risk subgroup, whereas the immune cell scores tended to be lower in the high-risk subgroup. In the MUC training set, the high-risk subgroup showed high immune cell infiltration scores in the following cell types: Treg cells, Th2 cells, macrophages, macrophages M1, macrophages M2, CD4^+^ T cells, CD4^+^ central memory T cells (CD4 + Tcm). In contrast, naive B cells, CD8^+^ T cells, and CD8^+^ central memory T cells (CD8 + Tcm) remained at a relatively low level in the high-risk subgroup.

In the TCGA BLCA cohort, significantly decreased expression of naive CD4^+^ T cells, CD4^+^ central memory T cells (CD4 + Tcm), and naive CD8^+^ T cells was observed in the high-risk subgroup, while the expression of CD8 T cells, CD8^+^ central memory T cells (CD8 + Tcm), Th2 cells, and macrophages M1 tended to be low in the low-risk subgroup. In the GSE120736 bladder cancer cohort, B cells and CD4^+^ central memory T cells (CD4 + Tcm) were lower in the high-risk subgroup than in the low-risk subgroup. In comparison, the number of natural killer T (NKT) cells was high in the high hypoxia risk score subgroup (Figures 5A–D). The differentially expressed genes were HLA-DPA1, HLA-DPA2, BTN3A2, GZMA, CD27, PDCD1, and TIGIT (Supplementary Figure S5A).

### GSEA Analysis and Functional Verification

**FIGURE 6:**
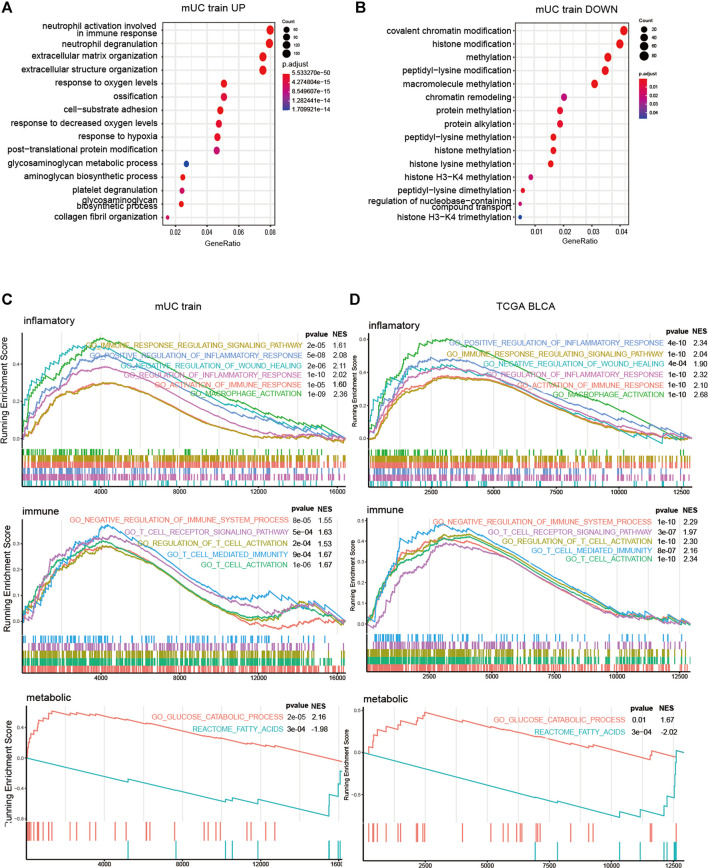
The results of GSEA analysis of risk score group. **(A,B)** Histogram showed the enrichment of up-regulated genes (*p* < 0.05, logFC > 0) and down regulated genes (*p* < 0.05, logfc < 0) in the top15 pathways of the GO gene set (*p* < 0.05, logFC < 0). The pathways were mainly the intercellular and hypoxia related pathways. **(C,D)** Results of GSEA analysis on inflammatory pathway, immune pathway, glucose metabolism and lipid metabolism in metastatic urothelial carcinoma (FigC) and TCGA BLCA (FigD) cohort (*p* < 0.05).

Next, we performed Gene Ontology (GO) analysis and GSEA analysis to elucidate the biochemical functions. We selected differentially expressed genes (DEGs) in the high-risk groups compared with low-risk groups (*p* < 0.05, logFC > 0) for a GO pathway enrichment analysis. In terms of the top15 GO pathways, the high-risk subgroup was mainly concentrated in the extracellular matrix remodeling related pathways (Figures 6A,B).

**FIGURE 7:**
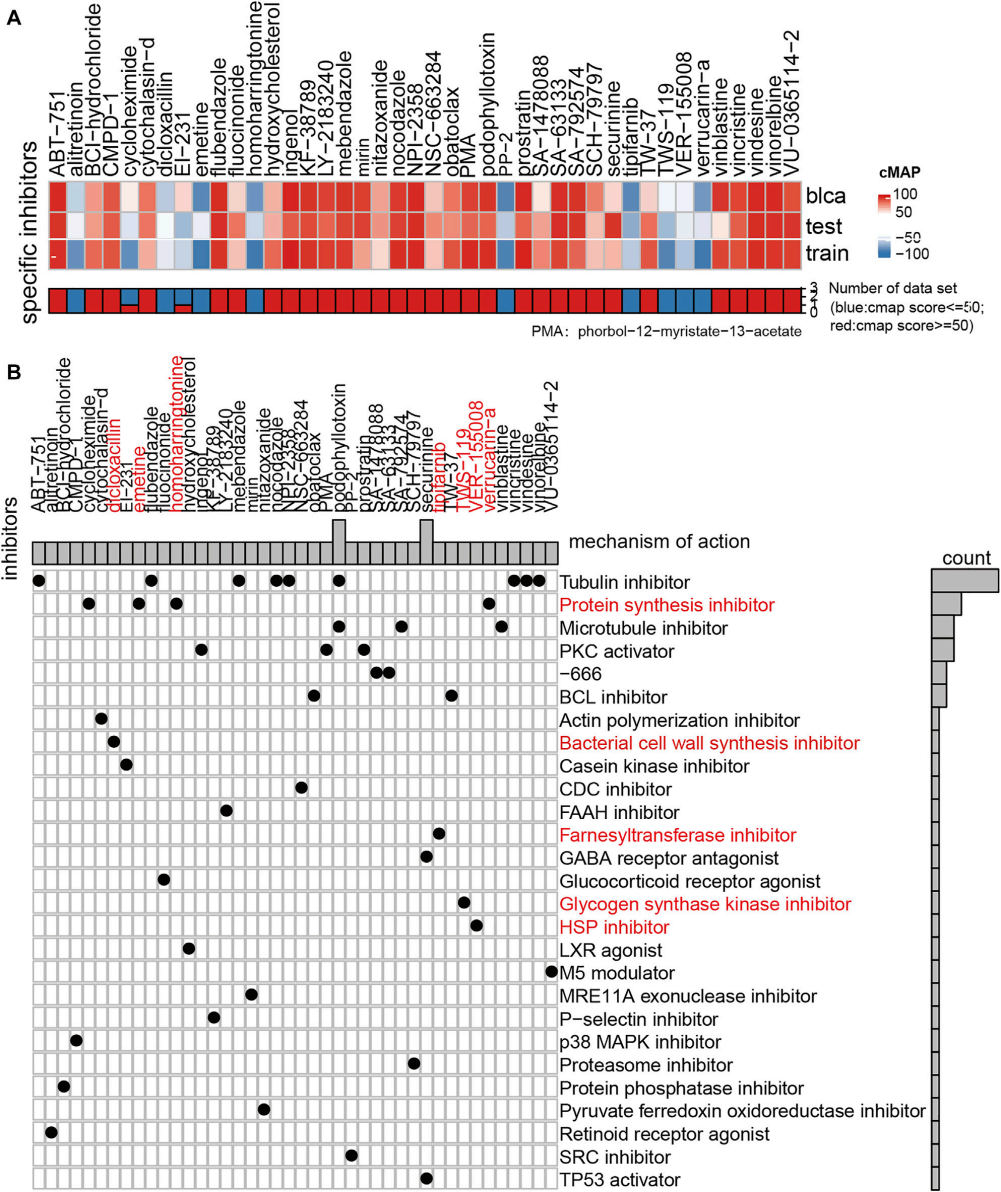
Results of drug sensitivity analysis of GDSC and CMAP. **(A)** Thermogram showed the small molecule drugs in the CMAP analysis results. Blue means that the cell expression profile treated with the small molecule drugs has similar effect with the low hypoxia score subgroup, and red represents that the effect of the cell expression profile treated with the small molecule drugs is similar to that of the high hypoxia score subgroup (|score| > = 60). **(B)** The drug molecular mechanism of small molecule drugs according to the above CMAP results.

To gain further insight into the potential mechanisms, we used GSEA to explore the relationship between hypoxia microenvironment and lipid metabolism, glucose metabolism, and immune pathways. In terms of metabolic pathways, the genes of the high-risk subgroup were mainly enriched in the glucose metabolism-related pathway. Meanwhile, the genes of the low-risk subgroup were mainly concentrated in lipid metabolism-related pathways. T cells, macrophages, and NK cells were significantly enriched in the high-risk subgroup in the immune process-related pathways but not in the low-risk subgroup. Meanwhile, inflammation-related pathways were enriched considerably in the high-risk subgroup (Figures 6C,D).

To further verify the associated signaling pathways activated in hypoxia microenvironment, we downloaded the GSE158632 dataset and classified six samples from the hypoxia group into the high-risk group and the other three samples from the control group into the low-risk group. Then, we performed GO analysis comparing the high- and low-risk groups. As is shown in Supplementary Figures S8A,C, RNA and mRNA catabolic processes were activated under hypoxia in the CACO2 cell line. Similarly, the GO analysis demonstrated that the hypoxia microenvironment was significantly associated with cellular response to topologically incorrect protein and regulation of autophagy in the HT29 cell line. These results indicated that the low level of oxygen during hypoxic conditions leading to increased abnormal protein expression and cell membrane instability of tumor cells.

Next, we calculated the activation degree of the glucose metabolic pathway through the GSEA algorithm to verify the role of glucose metabolism in the hypoxic tumor microenvironment. Pathway enrichment analysis indicated that glucose catabolic pathway was significantly enriched under hypoxic microenvironment both in the CACO2 and HT29 cell lines. This result was in consistent to a previous study from Denko (2008). Immune-related genes were reported to orchestrate tumor-associated immune responses; therefore, we further investigated the differences in the expression of immune-related genes. As is shown in Supplementary Figure S8G, the expression of immune-related genes ACTN4, TMBIM6, DAZAP2 and CAMTA1 were significantly up-regulated in the hypoxic groups.

### Drug Sensitivity Analysis

To explore the drug sensitivity using our hypoxia risk stratification system, we conducted a drug sensitivity analysis of 138 small and medium molecular drugs through the GDSC database. Clinically, cisplatin, gemcitabine, and methotrexate are commonly used for chemotherapy in urothelial carcinoma. These drugs did not show superior drug sensitivity in the subgroup with high hypoxia scores (*p* > 0.05) (Supplementary Figure S1D).

We also used CMAP to analyze the similarities in gene expression profiles between other drugs and the MUC hypoxia risk score group. The results show that protein synthesis inhibitor, bacterial cell wall synthesis inhibitor, farnesyltransferase inhibitor, glycogen synthase kinase inhibitor, and HSP inhibitor were effective in patients with a high hypoxia score. These drugs may reverse the hypoxia microenvironment of patients and changing it into a state of mild hypoxia.

## Discussion

Metastatic urothelial carcinoma usually responds poorly to treatment with immunosuppressive agents. In this study, we speculate that the insensitivity of some MUCs to ICI may be related to the hypoxic microenvironment. Therefore, we screened the hypoxia-related genes to construct a risk model to evaluate hypoxia microenvironment and predict the survival of patients with MUC undergoing ICI treatment. We constructed a hypoxia risk prognostic model based on four hypoxia-related genes (TKTL1, JMJD6, IRS2, ANXA2) through multivariate Cox regression analysis. TKTL1 was negatively correlated with survival rate, while JMJD6, IRS2 and ANXA2 showed a positive correlation. The robustness of the proposed model was evaluated on multiple cohorts. The results showed that the model effectively identified distinct subgroups with different hypoxia risk, indicating that high hypoxia was correlated with poor prognosis. Simultaneously, the potential mechanisms of action were determined by multi-omics studies.

We found that TP53, Rb1, KDM6A, and TSC1 experience mutational inactivation in a hypoxic microenvironment by analyzing the mutations of driver genes. TP53 is a known tumor suppressor gene and expresses p53 protein to regulate the DNA repair system in cells under normal conditions (Zhang and Zhang, 2015). Rb1 is a known tumor suppressor gene that regulates cell cycle (Dyson, 2016). Arakawa et al. found that Rb1 mutational inactivation may cause lung cancer resistance to PD-1 inhibitors (Arakawa et al., 2021). Our study found that Rb1 and TP53 co-mutate in a hypoxic microenvironment, suggesting that Rb1 and TP53 mutational inactivation may cause tumor resistance to ICIs in a hypoxic microenvironment (Ku et al., 2017). KDM6A belongs to the KDM6 family of histone H3 lysine 27 (H3K27) demethylases. KDM6A deletion mutation induce a repressive H3K27 demethylation state and block differentiation (Chakraborty et al., 2019). Yuichiro et al. (Itoh et al., 2019) found that a KDM6A deletion mutation resulted in the activation of Th1 and Th2 cell pathways and the down regulation of an inflammatory response in CD4^+^ T cells, which is likely to contribute to a pro-tumor microenvironment. Thus, we consider that a hypoxic microenvironment may promotes the formation of KDM6A deletion mutation, enhancing the differentiation of immunosuppressive cells, and ultimately results in immunological resistance and poor prognosis. Our mutation analysis also found that KDM6A and FGFR3 have co-occurrence, suggesting that both may lead to immune resistance. The protein encoded by TSC1 and TSC2 constitutes tuberous sclerosis (TSC) complex, which negatively impacts the regulation of mTOR activity, and participates in the functional regulation of macrophages (Yang et al., 2011; Yang et al., 2013). Meanwhile, tumor-associated macrophages tend to differentiate into M2 type and inhibit immune response in a hypoxic microenvironment (Samanta and Semenza, 2018). Therefore, unique driver gene mutations in a hypoxic microenvironment may enhance the ability of tumor cells to escape being killed by immune cells or affect the function of immune-related cells. This may lead to a poor response to immunosuppressive therapy.

The hypoxia microenvironment facilitates glucose uptake in malignant tumor cells, therefore affects the functions of some of the most important immunologically active cells, especially the recognition and clearance functions of T cells. We found that the infiltration of CD4^+^ T cells in MUC was slightly higher than in the high-risk subgroup. Treg cells, macrophages, and Th2 cells play an inhibitory role in ICI treatment, also have a higher infiltration degree in the high-risk subset (Gajewski et al., 2013; Samanta and Semenza, 2018). The research of Chang et al. (2015) shows that tumor cells compete with T cells for glucose in a mouse model, and the ability of tumor cells to utilize glucose for energy is more potent than that of T cells, which results in the inhibition of nutritional metabolism in T cells. Hypoxia increases the activation of glucose metabolism-related pathways as well as the competitive uptake of glucose by tumor cells, which promotes T cells to a unfavorable nutritional status and inhibits T cells from carrying out their immune functions or clearing out tumor cells. Additionally, the enhanced glycolysis activity of tumor cells in a hypoxic environment leads to an acidic microenvironment, which also affects the function of T cells (Leone and Powell, 2020). The PDCD1 gene was up-regulated in the high hypoxia risk subgroup. PDCD1 encodes PD-1 protein, an essential protein on the surface of T cells (and other immune cells) that recognizes abnormal cells. It has been found that in an inflammatory environment, PD-1 protein is overexpressed on the surface of T cells. This overexpression inhibits the expression of PD-1 protein in T cells around the tumor and suppresses a T cell-mediated immune response (Baumeister et al., 2016; Multhoff and Vaupel, 2020). In addition, Nicole E. et al. established a mouse model with a hypoxia microenvironment to observe possible changes in T cells under hypoxic conditions (Scharping et al., 2021). Their research confirmed that the metabolic stress under hypoxia could accelerate the terminal cell differentiation, increase reactive oxygen species (ROS) level of T cells, and eventually cause severe T-cell dysfunction, which further verified our conjecture.

One of the crucial mechanisms of tumor invasion and metastasis mediated by a hypoxic microenvironment is the degradation of fibrin and collagen (Ricard-Blum, 2011; Dragoš and Kovács, 2017). The results of the GO analysis showed that genes related to extracellular matrix remodeling exist in the subgroup with a high hypoxia score. The destruction of stromal cells can easily lead to abnormal differentiation and malfunction of T cells, resulting in immunosuppression (Vito et al., 2020). Therefore, we believe that hypoxia can promote the decomposition and reconstruction of the matrix structure of tumor cells. Additionally, it may inhibit the differentiation of T cells into Th1 cells and promote the differentiation of T cells into Treg cells, thus leading to immunosuppression (Schito and Semenza, 2016).

Several limitations to the present study should be considered. First, although we have included as many ICI treatment cohorts as possible to verify the accuracy of the model, the sample size of some cohort is small. In addition, the validation cohort used is based on retrospective data, so the model needs to be further validated in large-scale clinical studies. Finally, the theoretical mechanisms need to be further verified by biological experiments.

## Conclusion

Our study establishes a novel four-gene risk stratification system that could inform ICIs treatment response in urothelial carcinoma with by evaluating hypoxic microenvironment. In addition, we systematically explained the reasons for the poor efficacy of ICI treatment in MUC with a hypoxic microenvironment from the perspectives of genome, transcriptome sequencing data, and the immune microenvironment. The validation of our results and the underlying mechanisms remain to be studied further.

## Data Availability

The datasets supporting the conclusions of this article are available in the R package "IMvigor210CoreBiologies" (http://research-pub.gene.com/IMvigor210CoreBiologies), and GEOdatabase (https://www.ncbi.nlm.nih.gov/geo/query/acc.cgi?acc=GSE120736, https://www.ncbi.nlm.nih.gov/geo/query/acc.cgi?acc=GSE78220, https://www.ncbi.nlm.nih.gov/geo/query/acc.cgi?acc=GSE158632).
