# Supplementary material for: Hypoxic Characteristic Genes Predict Response to Immunotherapy for Urothelial Carcinoma
Source: Front Cell Dev Biol. 2021 Nov 25;9:762478. doi: 10.3389/fcell.2021.762478 (PMC8657403; doi:10.3389/fcell.2021.762478)
Supplement: Supplementary file 5 [file DataSheet1.PDF]

Supplementary Material

1 Supplementary Figures and Tables

1.1 Supplementary Figures

A

B

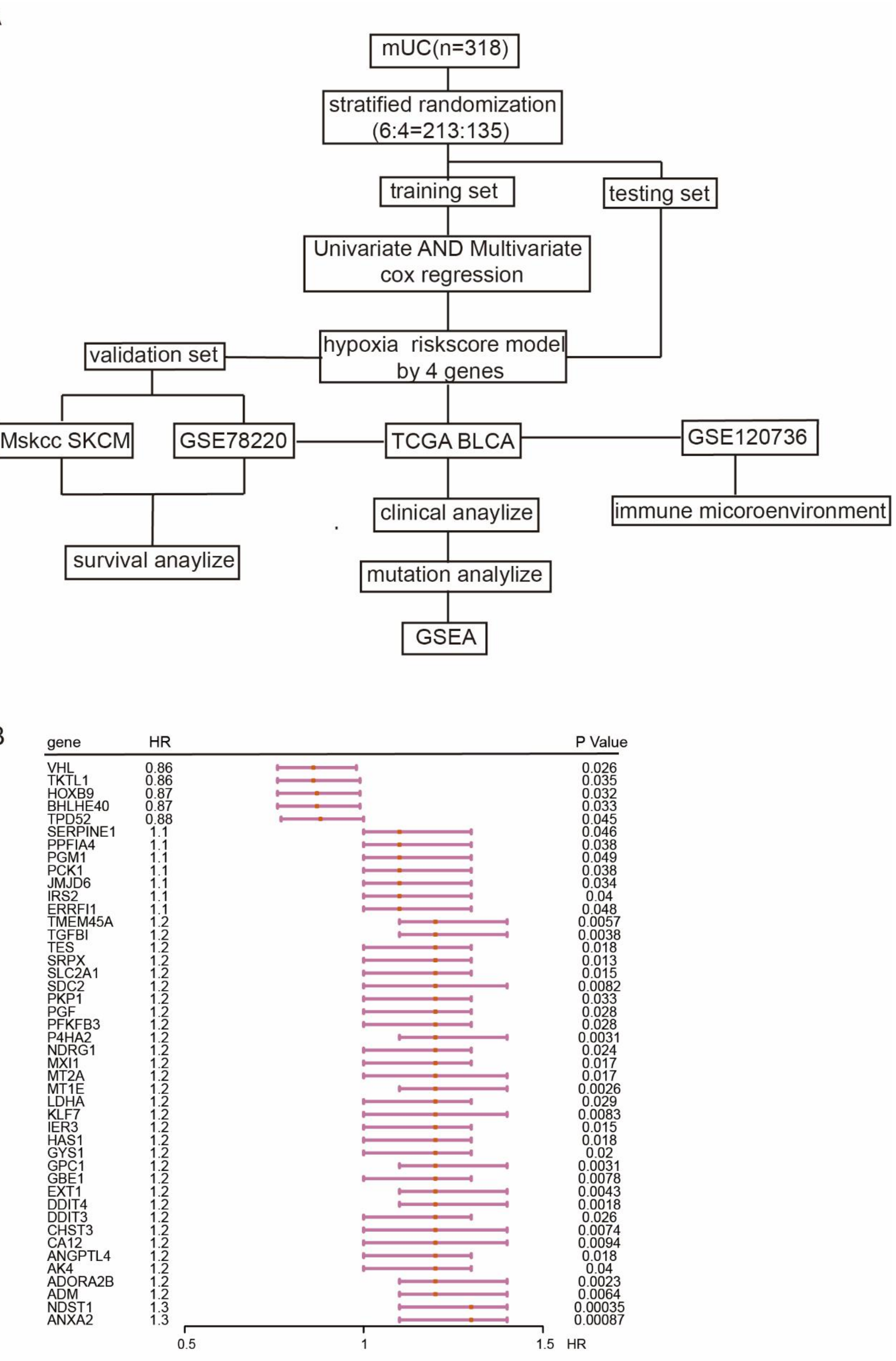
mUC(n=318)
stratified randomization (6:4=213:135)
training set
testing set
Univariate AND Multivariate cox regression
hypoxia riskscore model by 4 genes
validation set
Mskcc SKCM
GSE78220
TCGA BLCA
GSE120736
clinical anaylize
immune micoroenvironment
survival anaylize
mutation analylize
GSEA
gene HR P Value
VHL 0.86 0.026
TKTL1 0.86 0.035
HOXB9 0.87 0.032
BHLHE40 0.87 0.033
TPD52 0.88 0.045
SERPINE1 1.1 0.046
PPFIA4 1.1 0.038
PGM1 1.1 0.049
PCK1 1.1 0.038
JMJD6 1.1 0.034
IRS2 1.1 0.04
ERRFI1 1.1 0.048
TMEM45A 1.2 0.0057
TGFBI 1.2 0.0038
TES 1.2 0.018
SRPX 1.2 0.013
SLC2A1 1.2 0.015
SDC2 1.2 0.0082
PKP1 1.2 0.033
PGF 1.2 0.028
PFKFB3 1.2 0.028
P4HA2 1.2 0.0031
NDRG1 1.2 0.024
MXI1 1.2 0.017
MT2A 1.2 0.017
MT1E 1.2 0.0026
LDHA 1.2 0.029
KLF7 1.2 0.0083
IER3 1.2 0.015
HAS1 1.2 0.018
GYS1 1.2 0.02
GPC1 1.2 0.0031
GBE1 1.2 0.0078
EXT1 1.2 0.0043
DDIT4 1.2 0.0018
DDIT3 1.2 0.026
CHST3 1.2 0.0074
CA12 1.2 0.0094
ANGPTL4 1.2 0.018
AK4 1.2 0.04
ADORA2B 1.2 0.0023
ADM 1.2 0.0064
NDST1 1.3 0.00035
ANXA2 1.3 0.00087
0.5
1
1.5
HR

Supplementary Figure 1. (A) Flow chart of the construction and analysis of the prognosis model of hypoxia risk score. (B) The forest map showed that 44 hypoxia related genes were obtained by univariate Cox regression.

A

mUC train

charactertics	high (N=106)	low (N=107)	Total (N=213)
**Sex**			
Male	24 (22.6%)	21 (19.6%)	45 (21.1%)
Female	82 (77.4%)	86 (80.4%)	168 (78.9%)
**Age (months)**			
(less than) 1 year	62 (58.5%)	44 (41.1%)	106 (49.8%)
1-2 years	23 (21.7%)	38 (35.5%)	61 (28.6%)
more than 2 years	21 (19.8%)	25 (23.4%)	46 (21.6%)
**censOS**			
Dead	87 (82.1%)	54 (50.5%)	141 (66.2%)
Alive	19 (17.9%)	53 (49.5%)	72 (33.8%)
**TC Level**			
TC0	73 (68.9%)	93 (86.9%)	166 (77.9%)
TC1	10 (9.4%)	6 (5.6%)	16 (7.5%)
TC2+	23 (21.7%)	7 (6.5%)	30 (14.1%)
Missing	0 (0%)	1 (0.9%)	1 (0.5%)
**binaryResponse**			
CR/PR	10 (9.4%)	33 (30.8%)	43 (20.2%)
SD/PD	82 (77.4%)	57 (53.3%)	139 (65.3%)
Missing	14 (13.2%)	17 (15.9%)	31 (14.5%)

mUC test

charactertics	high (N=67)	low (N=68)	Total (N=135)
**Sex**			
Male	12 (17.9%)	19 (27.9%)	31 (23.0%)
Female	55 (82.1%)	49 (72.1%)	104 (77.0%)
**Age (months)**			
(less than) 1 year	42 (62.7%)	28 (41.2%)	70 (51.9%)
1-2 years	18 (26.9%)	20 (29.4%)	38 (28.1%)
more than 2 years	7 (10.4%)	20 (29.4%)	27 (20.0%)
**censOS**			
Dead	51 (76.1%)	40 (58.8%)	91 (67.4%)
Alive	16 (23.9%)	28 (41.2%)	44 (32.6%)
**TC Level**			
TC0	51 (76.1%)	58 (85.3%)	109 (80.7%)
TC1	5 (7.5%)	1 (1.5%)	6 (4.4%)
TC2+	11 (16.4%)	9 (13.2%)	20 (14.8%)
**binaryResponse**			
CR/PR	9 (13.4%)	16 (23.5%)	25 (18.5%)
SD/PD	44 (6 .7%)	47 (69.1%)	91 (67.4%)
Missing	14 (20.9%)	5 (7.4%)	19 (14.1%)

Supplementary Figure 2. On the left side is a three line table of the mUC training set, and on the right side is a three line table of the mUC testing set that shows the distribution of clinical characteristics in the risk score subgroups

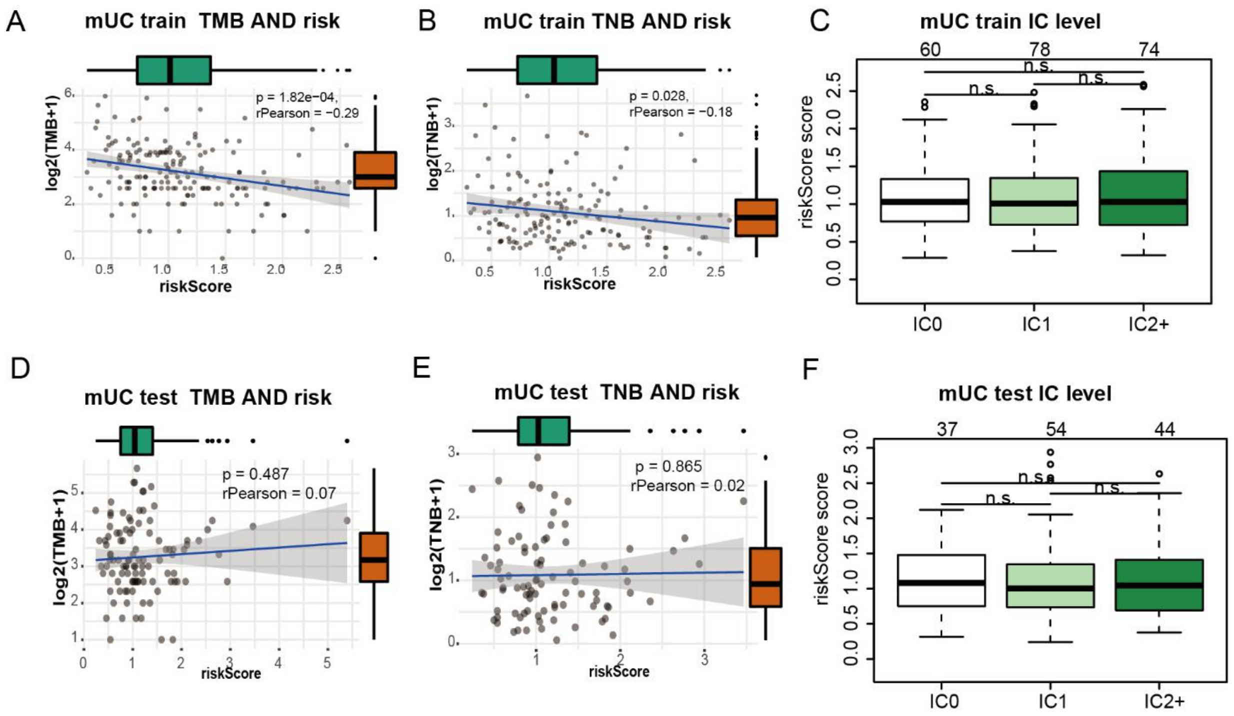

Supplementary Figure 3. (A) and (B) The correlation diagram shows the relationship between mUC training set risk score, TMB and TNB. (C) Box plot showed the expression of mUC training set risk score and immune cells PD-L1 expression. (D) and (E) Correlation diagram shows the correlation between mUC testing set risk score, TMB and TNB. (F) Box plot showed the immune cells PD-L1 expression of the mUC testing set and the relation to risk score

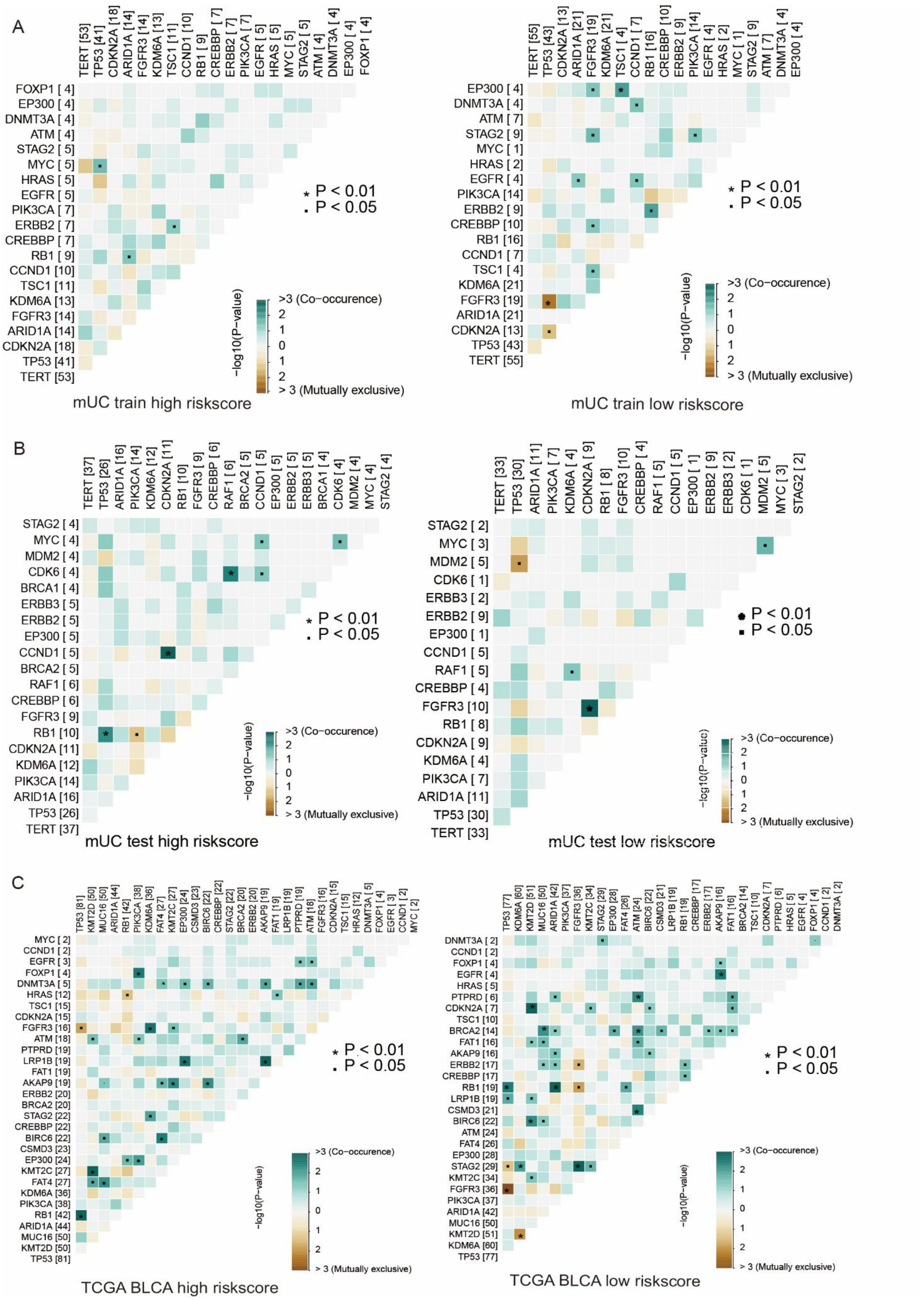

A
FOXP1 [4]
EP300 [4]
DNMT3A [4]
ATM [4]
STAG2 [5]
MYC [5]
HRAS [5]
EGFR [5]
PIK3CA [7]
ERBB2 [7]
CREBBP [7]
RB1 [9]
CCND1 [10]
TSC1 [11]
KDM6A [13]
FGFR3 [14]
ARID1A [14]
CDKN2A [18]
TP53 [41]
TERT [53]
* P < 0.01
. P < 0.05
-log10(P-value)
>3 (Co-occurence)
> 3 (Mutually exclusive)
mUC train high riskscore
EP300 [4]
DNMT3A [4]
ATM [7]
STAG2 [9]
MYC [1]
HRAS [2]
EGFR [4]
PIK3CA [14]
ERBB2 [9]
CREBBP [10]
RB1 [16]
CCND1 [7]
TSC1 [4]
KDM6A [21]
FGFR3 [19]
ARID1A [21]
CDKN2A [13]
TP53 [43]
TERT [55]
* P < 0.01
. P < 0.05
mUC train low riskscore
B
STAG2 [4]
MYC [4]
MDM2 [4]
CDK6 [4]
BRCA1 [4]
ERBB3 [5]
ERBB2 [5]
EP300 [5]
CCND1 [5]
BRCA2 [5]
RAF1 [6]
CREBBP [6]
FGFR3 [9]
RB1 [10]
CDKN2A [11]
KDM6A [12]
PIK3CA [14]
ARID1A [16]
TP53 [26]
TERT [37]
* P < 0.01
. P < 0.05
mUC test high riskscore
STAG2 [2]
MYC [3]
MDM2 [5]
CDK6 [1]
ERBB3 [2]
ERBB2 [9]
EP300 [1]
CCND1 [5]
RAF1 [5]
CREBBP [4]
FGFR3 [10]
RB1 [8]
CDKN2A [9]
KDM6A [4]
PIK3CA [7]
ARID1A [11]
TP53 [30]
TERT [33]
P < 0.01
P < 0.05
mUC test low riskscore
C
* P < 0.01
. P < 0.05
TCGA BLCA high riskscore
TCGA BLCA low riskscore

Supplementary Figure 4. (A) (B) and (C) Correlation diagram shows the co-occurrence and mutually exclusive analysis results of the driver genes involved in the panorama of the mutant genes in the mUC training set, mUC testing set and TCGA BLCA cohort (* means P < 0.05, · represents P < 0.05).

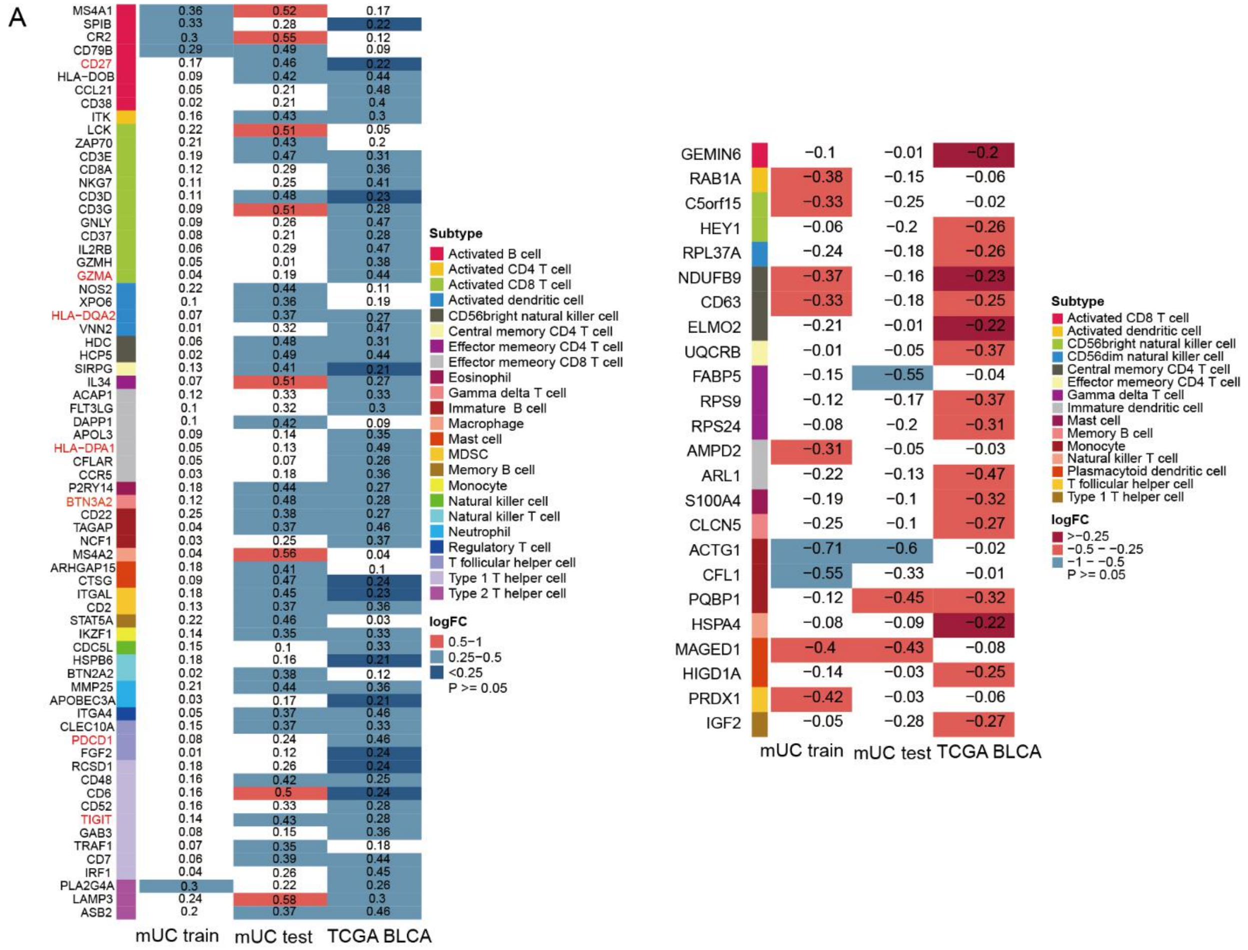

Supplementary Figure 5. Rankheatmap shows the up-regulated and down-regulated differentially expressed genes in the mUC training set, testing set and TCGA BLCA of metastatic urothelial carcinoma. Results show that antigen presentation related MHC factors included HLA-DPA1 and HLA-DPA2, Immune stimulator included BTN3A2, GZMA, CD27, Immunosuppressive factors included PDCD1, TIGIT are differentially expressed.

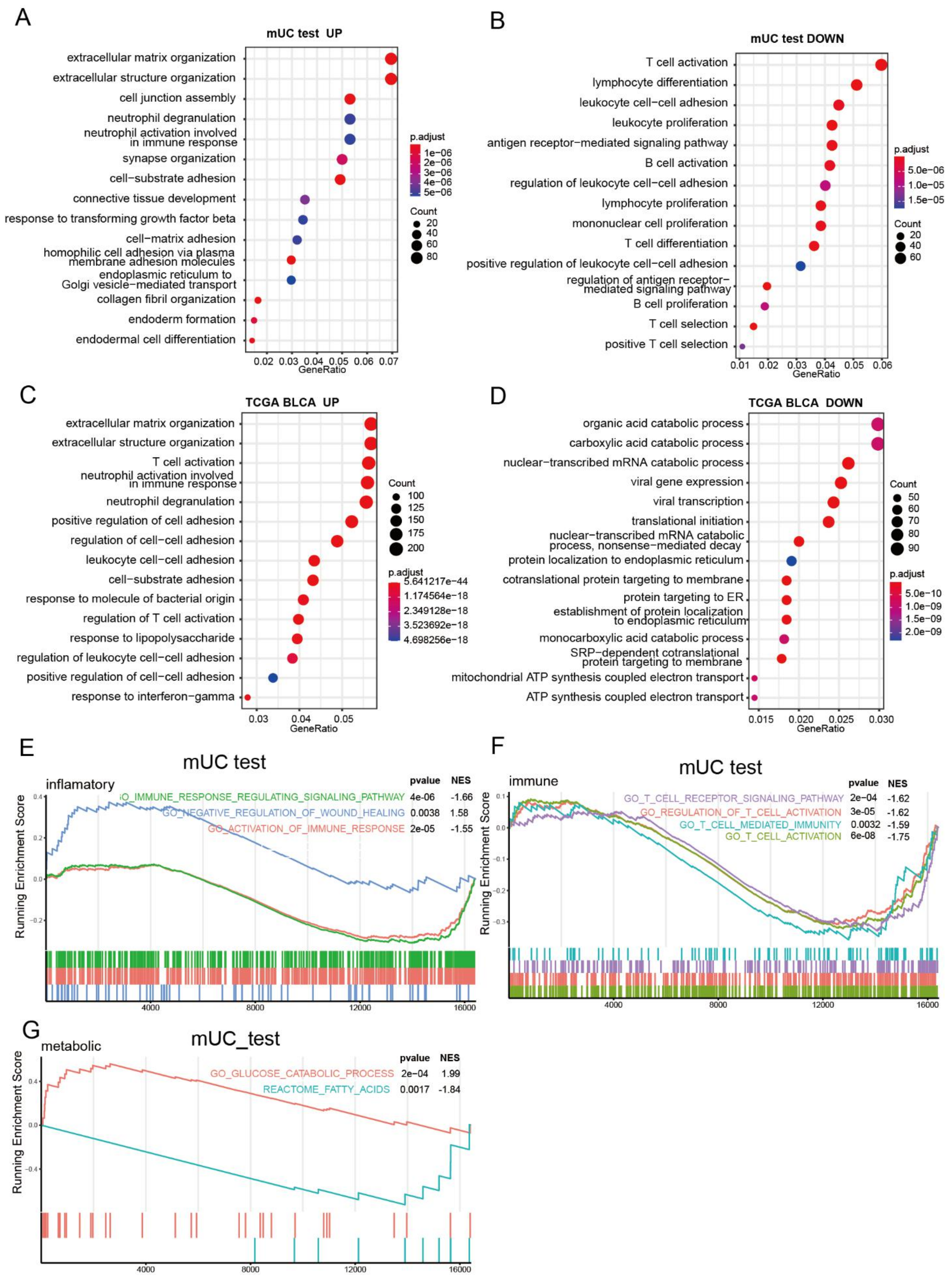

A
mUC test UP
extracellular matrix organization
extracellular structure organization
cell junction assembly
neutrophil degranulation
neutrophil activation involved in immune response
synapse organization
cell−substrate adhesion
connective tissue development
response to transforming growth factor beta
cell−matrix adhesion
homophilic cell adhesion via plasma membrane adhesion molecules
endoplasmic reticulum to Golgi vesicle−mediated transport
collagen fibril organization
endoderm formation
endodermal cell differentiation
GeneRatio
p.adjust
1e−06
2e−06
3e−06
4e−06
5e−06
Count
20
40
60
80
B
mUC test DOWN
T cell activation
lymphocyte differentiation
leukocyte cell−cell adhesion
leukocyte proliferation
antigen receptor−mediated signaling pathway
B cell activation
regulation of leukocyte cell−cell adhesion
lymphocyte proliferation
mononuclear cell proliferation
T cell differentiation
positive regulation of leukocyte cell−cell adhesion
regulation of antigen receptor−mediated signaling pathway
B cell proliferation
T cell selection
positive T cell selection
GeneRatio
p.adjust
5.0e−06
1.0e−05
1.5e−05
Count
20
40
60
C
TCGA BLCA UP
extracellular matrix organization
extracellular structure organization
T cell activation
neutrophil activation involved in immune response
neutrophil degranulation
positive regulation of cell adhesion
regulation of cell−cell adhesion
leukocyte cell−cell adhesion
cell−substrate adhesion
response to molecule of bacterial origin
regulation of T cell activation
response to lipopolysaccharide
regulation of leukocyte cell−cell adhesion
positive regulation of cell−cell adhesion
response to interferon−gamma
GeneRatio
Count
100
125
150
175
200
p.adjust
5.641217e−44
1.174564e−18
2.349128e−18
3.523692e−18
4.698256e−18
D
TCGA BLCA DOWN
organic acid catabolic process
carboxylic acid catabolic process
nuclear−transcribed mRNA catabolic process
viral gene expression
viral transcription
translational initiation
nuclear−transcribed mRNA catabolic process, nonsense−mediated decay
protein localization to endoplasmic reticulum
cotranslational protein targeting to membrane
protein targeting to ER
establishment of protein localization to endoplasmic reticulum
monocarboxylic acid catabolic process
SRP−dependent cotranslational protein targeting to membrane
mitochondrial ATP synthesis coupled electron transport
ATP synthesis coupled electron transport
GeneRatio
Count
50
60
70
80
90
p.adjust
5.0e−10
1.0e−09
1.5e−09
2.0e−09
E
mUC test
inflamatory
pvalue NES
GO_IMMUNE_RESPONSE_REGULATING_SIGNALING_PATHWAY 4e-06 -1.66
GO_NEGATIVE_REGULATION_OF_WOUND_HEALING 0.0038 1.58
GO_ACTIVATION_OF_IMMUNE_RESPONSE 2e-05 -1.55
Running Enrichment Score
F
mUC test
immune
pvalue NES
GO_T_CELL_RECEPTOR_SIGNALING_PATHWAY 2e−04 -1.62
GO_REGULATION_OF_T_CELL_ACTIVATION 3e-05 -1.62
GO_T_CELL_MEDIATED_IMMUNITY 0.0032 -1.59
GO_T_CELL_ACTIVATION 6e-08 -1.75
Running Enrichment Score
G
mUC_test
metabolic
pvalue NES
GO_GLUCOSE_CATABOLIC_PROCESS 2e−04 1.99
REACTOME_FATTY_ACIDS 0.0017 -1.84
Running Enrichment Score

Supplementary Figure 6. (A) and (B) The bubble chart showed the enrichment of up-regulated genes ($P < 0.05$, $logFC > 0$) and down regulated genes in the top 15 pathway of the GO gene set ($P < 0.05$, $logFC < 0$) with high vs low hypoxia risk score in the mUC testing set cohort ($P < 0.05$, $logFC < 0$). (C) and (D) Bubble chart showed the enrichment of up-regulated genes ($P < 0.05$, $logFC > 0$) and down regulated genes in the top 15 pathway of the GO gene set ($P < 0.05$, $logFC < 0$) in TCGA BLCA cohort. (E) (F) and (G) GSEA analysis results of inflammatory pathway, immune pathway, glucose metabolism and lipid metabolism in metastatic urothelial carcinoma ($P < 0.05$).

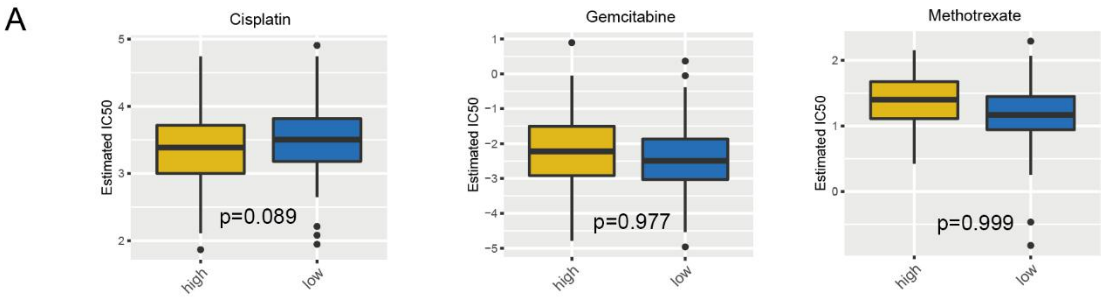

Supplementary Figure 7. Histogram of the sensitivity of common clinical chemotherapy drugs in the hypoxia risk score group. The three chemotherapeutic drugs, cisplatin, gemcitabine and methotrexate, showed in the chart did not show better drug sensitivity in the subgroup with low hypoxia score (P > 0.05).

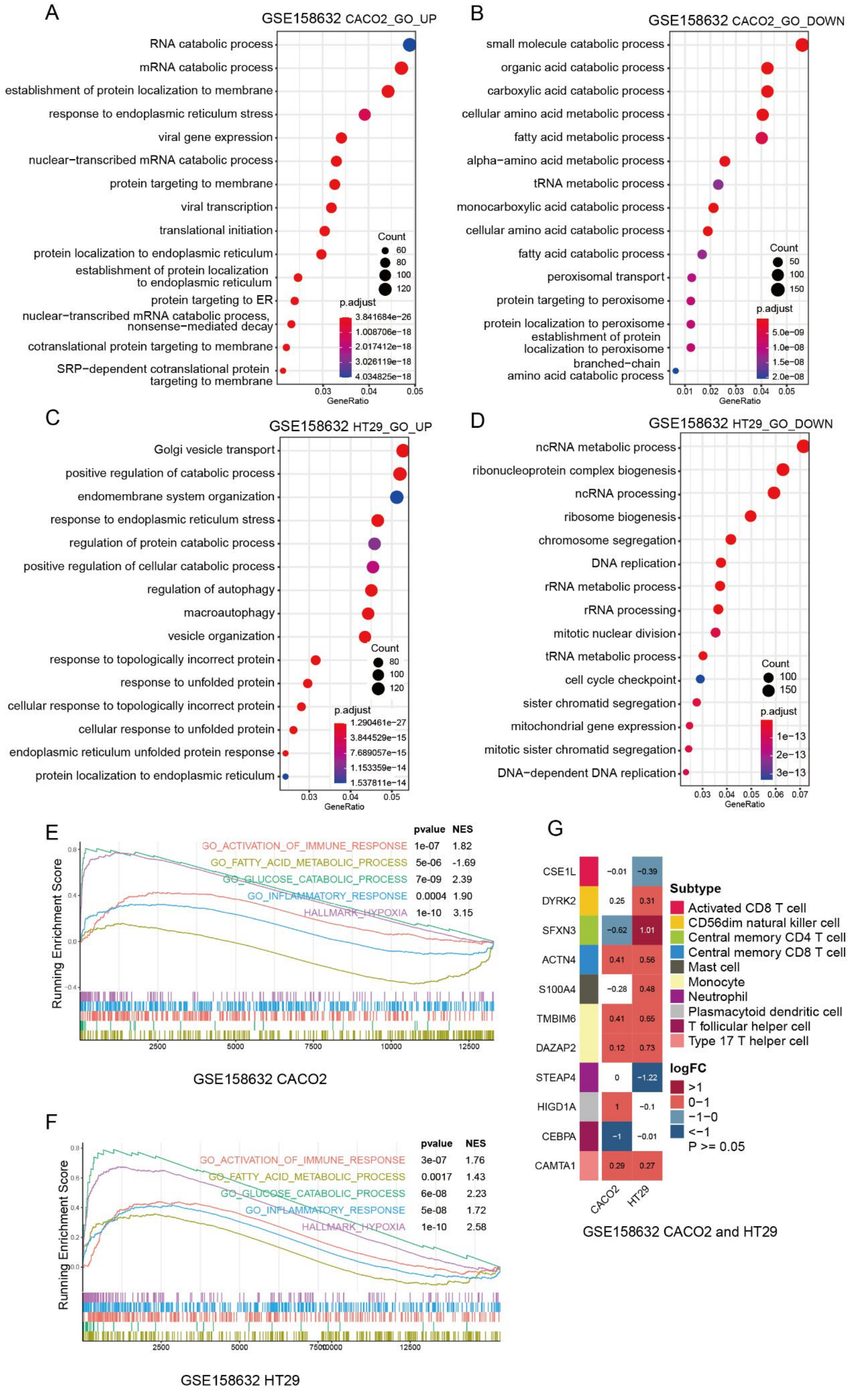

A
GSE158632 CACO2_GO_UP
RNA catabolic process
mRNA catabolic process
establishment of protein localization to membrane
response to endoplasmic reticulum stress
viral gene expression
nuclear−transcribed mRNA catabolic process
protein targeting to membrane
viral transcription
translational initiation
protein localization to endoplasmic reticulum
establishment of protein localization to endoplasmic reticulum
protein targeting to ER
nuclear−transcribed mRNA catabolic process, nonsense−mediated decay
cotranslational protein targeting to membrane
SRP−dependent cotranslational protein targeting to membrane
Count
60
80
100
120
p.adjust
3.841684e−26
1.008706e−18
2.017412e−18
3.026119e−18
4.034825e−18
0.03
0.04
0.05
GeneRatio
B
GSE158632 CACO2_GO_DOWN
small molecule catabolic process
organic acid catabolic process
carboxylic acid catabolic process
cellular amino acid metabolic process
fatty acid metabolic process
alpha−amino acid metabolic process
tRNA metabolic process
monocarboxylic acid catabolic process
cellular amino acid catabolic process
fatty acid catabolic process
peroxisomal transport
protein targeting to peroxisome
protein localization to peroxisome
establishment of protein localization to peroxisome
branched−chain amino acid catabolic process
Count
50
100
150
p.adjust
5.0e−09
1.0e−08
1.5e−08
2.0e−08
0.01
0.02
0.03
0.04
0.05
GeneRatio
C
GSE158632 HT29_GO_UP
Golgi vesicle transport
positive regulation of catabolic process
endomembrane system organization
response to endoplasmic reticulum stress
regulation of protein catabolic process
positive regulation of cellular catabolic process
regulation of autophagy
macroautophagy
vesicle organization
response to topologically incorrect protein
response to unfolded protein
cellular response to topologically incorrect protein
cellular response to unfolded protein
endoplasmic reticulum unfolded protein response
protein localization to endoplasmic reticulum
Count
80
100
120
p.adjust
1.290461e−27
3.844529e−15
7.689057e−15
1.153359e−14
1.537811e−14
0.03
0.04
0.05
GeneRatio
D
GSE158632 HT29_GO_DOWN
ncRNA metabolic process
ribonucleoprotein complex biogenesis
ncRNA processing
ribosome biogenesis
chromosome segregation
DNA replication
rRNA metabolic process
rRNA processing
mitotic nuclear division
tRNA metabolic process
cell cycle checkpoint
sister chromatid segregation
mitochondrial gene expression
mitotic sister chromatid segregation
DNA−dependent DNA replication
Count
100
150
p.adjust
1e−13
2e−13
3e−13
0.03
0.04
0.05
0.06
0.07
GeneRatio
E
pvalue NES
GO_ACTIVATION_OF_IMMUNE_RESPONSE 1e-07 1.82
GO_FATTY_ACID_METABOLIC_PROCESS 5e-06 -1.69
GO_GLUCOSE_CATABOLIC_PROCESS 7e-09 2.39
GO_INFLAMMATORY_RESPONSE 0.0004 1.90
HALLMARK_HYPOXIA 1e-10 3.15
Running Enrichment Score
0.8
0.4
0.0
-0.4
2500
5000
7500
10000
12500
GSE158632 CACO2
F
pvalue NES
GO_ACTIVATION_OF_IMMUNE_RESPONSE 3e-07 1.76
GO_FATTY_ACID_METABOLIC_PROCESS 0.0017 1.43
GO_GLUCOSE_CATABOLIC_PROCESS 6e-08 2.23
GO_INFLAMMATORY_RESPONSE 5e-08 1.72
HALLMARK_HYPOXIA 1e-10 2.58
Running Enrichment Score
0.8
0.6
0.4
0.2
0.0
2500
5000
7500
10000
12500
GSE158632 HT29
G
CSE1L −0.01 −0.39
DYRK2 0.25 0.31
SFXN3 −0.62 1.01
ACTN4 0.41 0.56
S100A4 −0.28 0.48
TMBIM6 0.41 0.65
DAZAP2 0.12 0.73
STEAP4 0 −1.22
HIGD1A 1 −0.1
CEBPA −1 −0.01
CAMTA1 0.29 0.27
CACO2
HT29
Subtype
Activated CD8 T cell
CD56dim natural killer cell
Central memory CD4 T cell
Central memory CD8 T cell
Mast cell
Monocyte
Neutrophil
Plasmacytoid dendritic cell
T follicular helper cell
Type 17 T helper cell
logFC
>1
0−1
−1−0
<−1
P >= 0.05
GSE158632 CACO2 and HT29

Supplementary Figure 8. (A) and (B) The bubble chart showed the enrichment of up-regulated genes (P < 0.05, logFC > 0) and down-regulated genes in the top 15 pathway of the GO gene set (P < 0.05, logFC < 0) with high vs low hypoxia risk score in the GSE158632 CACO2 cells (P < 0.05, logFC < 0). (C) and (D) Bubble chart showed the enrichment of up-regulated genes (P < 0.05, logFC > 0) and down-regulated genes in the top 15 pathway of the GO gene set (P < 0.05, logFC < 0) in GSE158632 HT29 cells. (E) and (F) GSEA analysis results of inflammatory pathway, immune pathway, glucose metabolism and lipid metabolism in GSE158632 CACO2 and HT29 cells (P < 0.05). (G) Rankheatmap shows the up-regulated and down-regulated differentially expressed genes in the GSE158632 CACO2 and HT29 cells. Results show that ACTN4, TMBIM6, DAZAP2 and CAMTA1 are differentially expressed.

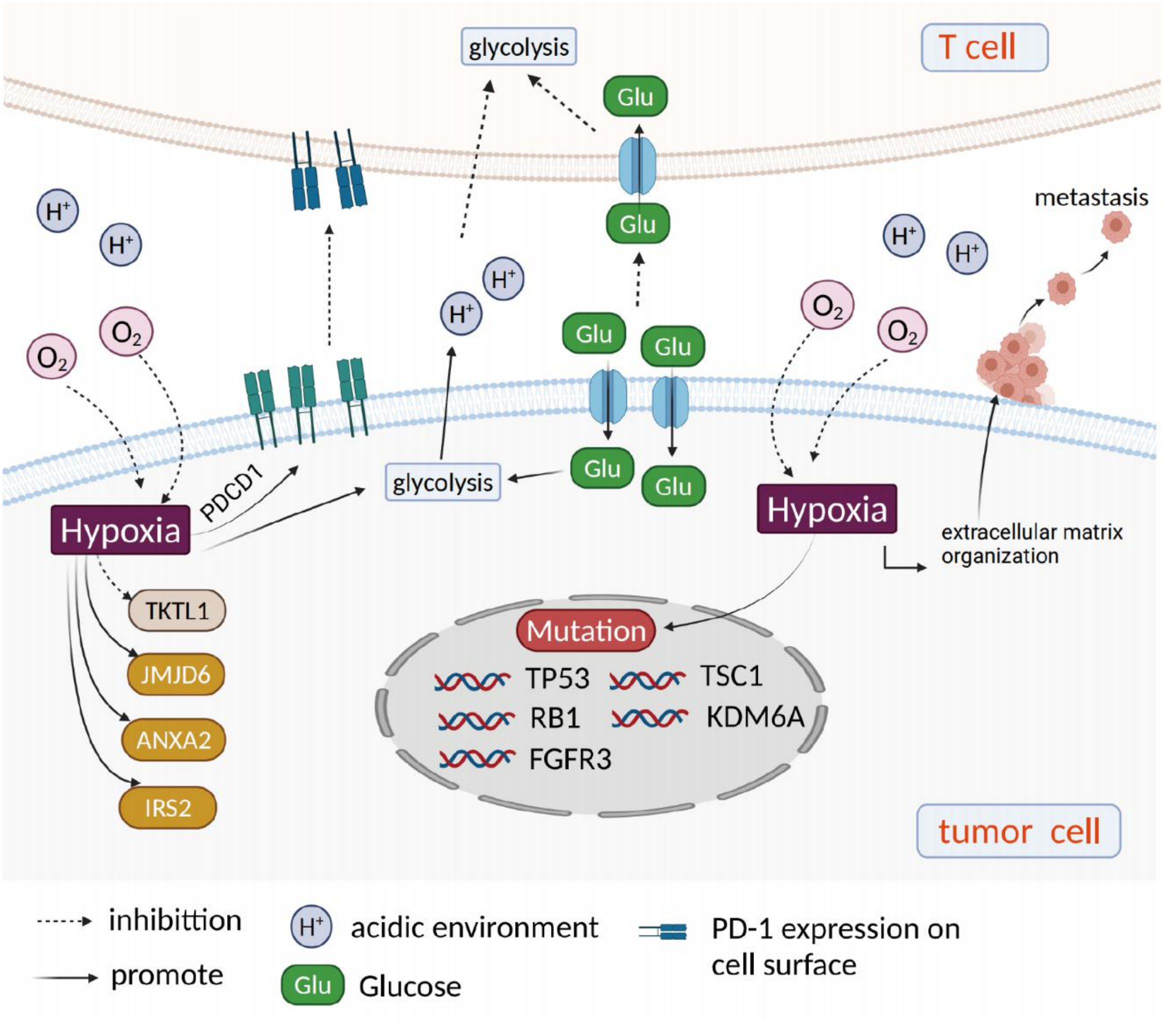

Supplementary Figure 9. Mechanism of hypoxia microenvironment inhibiting T cells. In the hypoxic microenvironment, tumor cells and T cells competitively ingest glucose for glycolysis, resulting in an acidic interstitial environment. In an acidic environment, the expression of PD-1 in tumor cells increases, suppressing the expression of PD-1 in T cells, thus inhibiting the function of T cells to recognize and kill tumors. Hypoxia promotes mesenchymal transformation of tumor cells,

making it easy for tumor cells to metastasize and evade the immune recognition of T cells. The inactivation mutations of Rb1, TP53, TSC1, KDM6A and FGFR3 in the hypoxia environment may be related to immune cell function and differentiation inhibition.

1.2 Supplementary Tables

Supplementary table 1,2,3,4. Transcriptome data, Mutation data or clinical data of metastatic urothelial cancer, bladder cancer, GSE78220 and Melanoma from MSKCC database. Count data was normalized using trimmed mean of M-values (TMM) and transformed with voom to log2-counts per million. Count data used for immune infiltration microenvironment was normalized and converted into the transcripts per million (TPM) data format.

Supplementary table 5. Risk score results of training set, testing set, TCGA BLCA, GSE78220, MSKCC SKCM datasets including overall survival time and status, gene expressions, and risk score degrees and Multivariate Cox regression results of the 4-gene hypoxia prognosis model including gene names, coefficients, and hazard ratios.

Supplementary table 6. Mutation matrixes, clinical information and results of mutual exclusion and co-occurrence analysis shown in Figure 4 and supplementary figure 4 of training set, testing set, TCGA BLCA datasets.

Supplementary table 7. Results of differential immune cells infiltration scores calculated by R package xCell of training set, testing set, TCGA BLCA and GSE120736 datasets.

Supplementary table 8. List of differential gene expression used for Gene Ontology analysis and Gene Set Enrichment Analysis (GSEA) of training set, testing set, TCGA BLCA datasets, GSE158632 CACO2 and HT29.

Supplementary table 9. List of immune related differential gene expression of training set, testing set, TCGA BLCA used for immune infiltration microenvironment analysis in supplementary Figure 5.

Supplementary table 10. Results of Gene Set Enrichment Analysis (GSEA) of training set, testing set, TCGA BLCA datasets, GSE158632 CACO2 and HT29.

Supplementary table 11. Results of Drug sensitivity analysis of training set, test set, TCGA BLCA datasets.
